# China’s science, technology, engineering, and mathematics (STEM) research environment: A snapshot

**DOI:** 10.1371/journal.pone.0195347

**Published:** 2018-04-03

**Authors:** Xueying Han, Richard P. Appelbaum

**Affiliations:** 1 Science and Technology Policy Institute, Washington DC, United States of America; 2 Center for Nanotechnology in Society, University of California at Santa Barbara, Santa Barbara, CA, United States of America; 3 Department of Global Studies, University of California at Santa Barbara, Santa Barbara, CA, United States of America; Iowa State University, UNITED STATES

## Abstract

In keeping with China’s President Xi Jinping’s “Chinese Dream,” China has set a goal of becoming a world-class innovator by 2050. China’s higher education Science, Technology, Engineering, and Math (STEM) research environment will play a pivotal role in influencing whether China is successful in transitioning from a manufacturing-based economy to an innovation-driven, knowledge-based economy. Past studies on China’s research environment have been primarily qualitative in nature or based on anecdotal evidence. In this study, we surveyed STEM faculty from China’s top 25 universities to get a clearer understanding of how faculty members view China’s overall research environment. We received 731 completed survey responses, 17% of which were from individuals who received terminal degrees from abroad and 83% of which were from individuals who received terminal degrees from domestic institutions of higher education. We present results on why returnees decided to study abroad, returnees’ decisions to return to China, and differences in perceptions between returnees and domestic degree holders on the advantages of having a foreign degree. The top five challenges to China’s research environment identified by survey respondents were: a promotion of short-term thinking and instant success (37% of all respondents); research funding (33%); too much bureaucratic or governmental intervention (31%); the evaluation system (27%); and a reliance on human relations (26%). Results indicated that while China has clearly made strides in its higher education system, there are numerous challenges that must be overcome before China can hope to effectively produce the kinds of innovative thinkers that are required if it is to achieve its ambitious goals. We also raise questions about the current direction of education and inquiry in China, particularly indications that government policy is turning inward, away from openness that is central to innovative thinking.

## Introduction

China’s transition from a manufacturing-based economy to an innovation-driven, knowledge-based economy relies heavily on advancements in science and technology (S&T) research and development (R&D). Whether China will be successful in overcoming the middle-income trap to become a high-income economy will largely depend on its ability to bridge the gap between basic research and commercialization. The research environment at Chinese institutions of higher education will, therefore, play a pivotal role in determining China’s trajectory. China’s central government understands the economic importance of having a vibrant, innovative research environment and has made giant strides over the past three decades to raise the overall level and quality of China’s research environment at institutions of higher education. The total number of Chinese universities approximately doubled from 1,792 to 2,560 institutions between 2005 and 2015 [1]; and roughly 1,200 of China’s higher education institutions are qualified to provide undergraduate B.A. degrees, with the others offering vocational or technical education [1, 2].

China has also observed a remarkable increase in the number of Science, Technology, Engineering, and Mathematics (STEM) graduates over the past three decades, providing the country with an abundant S&T workforce. In 2017, it was estimated that some 8 million students graduated from Chinese universities, a tenfold increase over a 20-year period, and twice the number graduating from U.S. universities [3]. Some 313,000 students were enrolled in doctoral programs in 2014, along with 1.5 million in M.A. programs; among these, 58 percent (182,000) were science or engineering Ph.D. students, and 44 percent (666,000) were science or engineering M.A. students [4]. China now boasts the largest number of laboratory scientists of any country [5], and its R&D spending outstrips that of the EU [6].

China’s increased investments in higher education and S&T R&D has led to a proliferation of scientific publications. China’s share of global scientific papers increased from 6.4% in 2003 to 18.2% in 2013, second only to the United States (18.8%) [7]. During this time period, China observed an 18.9% average annual growth rate in scientific papers, more than double that of the U.S. [7]. The quality of Chinese research publications, however, is another matter. Measuring quality of scientific output by percent share of a country’s scientific papers in the top 1 percent of papers cited worldwide, China’s progressing at a slower rate than it is publishing [7]. Although China’s share of scientific papers cited in the top 1 percent increased from 0.5% in 2002 to 0.8% in 2012, it remains well below that of the U.S. over the same time period (1.76% in 2002 and 1.94% in 2012) [7].

Despite China’s improvements in overall research output, there remain numerous challenges within its research environment that could prevent China from becoming the global leader in S&T that it wants to become. One recurring problem is academic fraud, in the form of faked peer review, discredited research resulting in journal articles that are later retracted, an extensive black market that provides everything from favorable peer reviews to published papers themselves, and the use of *guanxi* networks to secure favorable external evaluations of doctoral programs [1, 6]. Mismanagement (and sometimes corruption) has also been a problem in China’s highly touted foreign talents program, which provides generous incentives to bring foreign experts to China [8]. Additional challenges have to do with top-down governmental bureaucratization in the administration of higher education institutions; merit-based research funding, in which personal connections (*guanxi*) often take precedence over peer review; and a weak culture of performance evaluation which often emphasizes quantity over quality [9]. One approach the central government has taken to combat these challenges is through implementing initiatives such as the Thousand Talents Program and Young Thousand Talents Program [10], which prioritize bringing back prominent Chinese scholars, scientists, and entrepreneurs from abroad in hopes that they can help build China’s scientific capacity, and to facilitate the needed changes in China’s research environment. Whether these programs have been successful in bringing about the changes they were set up for has been a topic for debate but anecdotal evidence suggests that they have contributed to a new challenge within the research environment in which domestically trained scientists are discriminated against over returnees [9].

There have been many studies that have assessed these challenges to China’s research environment; however, most, if not all, are qualitative in nature [9, 11–17]; are based on case studies or a small sample size of interviews [17, 18]; or are primarily based on anecdotal evidence [19–26]. There are no studies, to our knowledge, that have taken a rigorous, systematic approach to evaluate China’s research environment and many basic questions remain unanswered. For example, what are the most pressing challenges to China’s research environment according to Chinese research faculty? What percent of Chinese faculty hold doctorate level degrees? What percent of Chinese faculty were trained abroad and what percent are home grown scholars? This study establishes a baseline for China’s research environment from which future research can build, and uses quantitative assessment to support claims that have only been made through anecdotal evidence.

## Methods

### Survey design

To systematically assess China’s scientific research environment, we attempted to contact all research faculty in STEM disciplines from the top 25 institutions of higher education. We initially sent surveys to more than 18,000 Chinese STEM faculty members at leading institutions of higher education. This study is based on findings from the 731 completed surveys that we received, the highest sample size, to our knowledge, in a study regarding China’s higher education research environment. STEM disciplines were limited to those identified by the U.S. Immigration and Customs Enforcement [27]. We identified the top 25 Chinese institutions of higher education by using WuShulian’s 2013 ranking of Chinese universities [28]. The ranking system uses a methodology similar to that of the Academic Ranking of World Universities [29]. There was originally some controversy regarding how the WuShulian rankings might have been biased as a result of bribery, with allegations made against Mr. Wu Shulian, who heads the ranking program and is a researcher at the Guangdong Chinese Academy of Management Science [29]. Despite these claims, the rankings by WuShulian remains one of the most detailed ranking systems as well as one of the oldest ranking systems, established in 1991, of Chinese institutions of higher education [30]. We also compared the 2013 WuShulian rankings against the 2013 and 2016 rankings issued by another well-known institution, the China Alumni Network [31, 32], and found that 24 out of 25 institutions listed by WuShulian were also in the top 25 listed by the China Alumni Network in 2013, and that 23 of institutions are still listed in the top 25 of the 2016 rankings by China Alumni Network (see S1 Appendix for university ranking and information). According to 2013 rankings, the 25 institutions in which we chose for our study are in the top 30 for both WuShulian and China Alumni Network. We are confident that these universities are representative of China’s top-tiered institutions of higher education. We also acknowledge that since our survey respondents are from first-tier institutions, they are unlikely to be representative of faculty members at second-tier and third-tier institutions. Therefore, the findings from our study likely represent the best-case scenarios of China’s higher education system and China’s research environment.

We collected contact information (i.e., email addresses) for all STEM research faculty by web scraping individual faculty profile pages from departmental websites. Some individuals provided multiple email addresses such that the total number of email addresses collected exceeded the total number of individuals. Contact information for 18,310 eligible individuals was collected using this method. After removal of duplicate addresses and those that no longer exist, we were left with a total of 18,163 eligible email addresses for our study. This study received human subjects approval from the University of California, Santa Barbara’s Office of Research Human Subjects Committee.

Individuals received an introductory letter via email detailing the purpose of the study, contact information for the authors and the UCSB Human Subjects Committee, and an anonymous link to participate in the survey (see S2 Appendix for the English and Chinese translations of the introductory letter). The survey was administered in Mandarin Chinese and distributed by Qualtrics (see S3 Appendix and S4 Appendix for the full survey translation in Mandarin Chinese and English, respectively). The first-round of surveys were sent during March 11–23, 2016. Weekly reminder emails were sent for a total of three weeks after the date of the initial email. Participants were given personalized links to access the survey such that once an individual completed the survey, the personalized link from the initial invitation email was no longer active. This was to prevent participants from sharing the survey with individuals who may not fall into our targeted population and to prevent participants from taking the survey more than once. During this first-round of surveys, 504 individuals started the survey, 374 completed it, and 8,996 email addresses (49.5% of all emails sent) were bounced back. We believe that the high number of bounce-backs were most likely due to China’s “Great Firewall.” In an effort to contact individuals whose email addresses bounced back, we used our personalized university email accounts to send the initial invitation letter in batches of 15–20 email addresses. From this effort, 181 individuals started the survey, and 129 completed it.

Because of the study’s low-response rate from the first-round of surveys, we decided to implement a second-round of surveys so that we could test for potential non-response bias. Individuals whose emails had not bounced and who had not completed the survey were contacted on August 17, 2016. They received two reminder emails at one-week intervals. A total of 180 completed surveys (out of the 235 started) were received from this second-round of surveys. And in a final attempt to increase the total number of responses to assess for non-response bias, we sent out a third-round of surveys on November 18, 2016, followed by two reminder emails at one-week intervals. This last effort resulted in a total of 48 completed surveys out of the 60 surveys that were started.

In sum, of the 18,163 email sent, we received a total of 503 completed surveys from the first-round of surveys and 228 completed surveys from the second- and third-round of surveys combined. This resulted in a total of 731 completed responses overall, yielding a 4.0% response rate.

### Non-response bias

Given the low response rate, we evaluated whether the study suffered from non-response bias and whether our survey responses could be generalized to the larger population of Chinese STEM faculty researchers at first-tiered institutions of higher education. We calculated non-response bias by categorizing respondents into two groups: (1) early respondents (individuals who completed the survey from the first-round of surveys); and (2) late respondents (individuals who completed the survey from the second- and third-round of surveys). By assuming late respondents are representative of non-respondents [33–37], we were able to calculate bias by examining the variation in responses, including demographics, between early and late respondents. Comparing early and late respondents is a common method to evaluate nonresponse bias and if survey results can be generalized to the larger intended population [38–40]. We chose this method for several reasons: it was feasible to achieve given the data that we had; the fact that there are no similar estimates that we can compare from other studies or sources; and that there are no comprehensive external database of the target population from which we can compare with our respondent population. We acknowledge that there are many different methods for assessing non-response bias, all of which have different strengths and weaknesses (for a review of the different methods, see [41]).

For single-answer questions, we cross-tabulated the response data and used Pearson’s chi-square test for independence to determine if there are significant differences in the distribution of responses between early and late respondents. In cases in which the total number of responses for each answer choice is too small (i.e., less than or equal to 5), Fisher’s exact test was used instead.

For multiple-answer questions, we used general linear models with a binomial distribution in which *time of response* was the independent variable and the dichotomous response for each answer choice (in which 0 indicated that the answer choice was not selected, and 1 indicated that the answer choice was selected) was the dependent variable. A type-III analysis of deviance was then used to evaluate whether *time of response* was a significant factor in each model. Chi-square and analysis of deviance results can be found in S5 Appendix for all multiple-choice questions. We found that with the exception of a few questions, there were no significant differences in the distribution of responses between early and late respondents. This suggests that our study does not suffer from non-response bias and that despite the low response rate, our results may be generalizable to the larger population. Given this finding, we decided to combine the responses from early and late respondents for all analyses.

### Summary statistics

Summary statistics (i.e., percent of respondents) were used to quantify degree attainment; if individuals held a foreign or domestic degree; where individuals studied abroad; if individuals remained in their host country to work after obtaining a Ph.D.; if individuals hold a dual faculty position in China and at a foreign institution; how long individuals remained overseas before returning to China; if returnees felt their foreign degree provided them with any advantages and if so, the types of advantages they have received; if domestic degree holders believed foreign degrees provided advantages and if so, what the perceived advantages are; why individuals studied abroad; why individuals returned to China; job satisfaction; departmental research culture satisfaction; field research culture satisfaction; and China research culture satisfaction.

For reasons to study abroad, respondents were able to select all reasons that applied. As a result, the percent of respondents across all choices add up to more than 100% and are not independent. We, therefore, used Cochran’s Q-test to determine whether the percent of respondents that selected each response differed across responses. Cochran’s Q-test is a non-parametric test that can be used to determine whether there are significant differences in frequencies (or proportions) across multiple dependent samples. We used the *cochran*.*qtest* function from the *RVAideMemoire* package (version 0.9-45-2) [42] in R to calculate Cochran’s Q. Pairwise comparisons using the Wilcoxon sign test were performed post-hoc to determine how responses differed significantly from one another.

To assess if returnees and domestic degree holders (i.e., homegrown scholars) differ in their perceptions on the types of advantages that are associated with having a foreign degree, we performed a two-sample proportion test for each advantage.

### Qualitative and statistical analyses

A total of 468 individuals responded to the free-response question “*What challenges (if any) do you think exist in China’s current research environment*?” Two responses were removed from all further analyses because their content did not address the question of interest. All responses were translated into English and each unique challenge to China’s scientific research environment was identified and recorded. Survey respondents pinpointed 42 unique challenges to China’s research environment. Each response was coded using the following scheme for all 42 challenges: 0 if the challenge was not identified in the response, 1 if the challenge was identified in the response.

Because many of the 42 challenges were similar in scope, we combined similar challenges into larger, more inclusive themes. Challenges were only grouped together when there were specific linkages identified by the respondents. For example, the larger theme of *research funding* is comprised of a subset of three individual challenges—*research funding is low*; *the funding process is unfair and non-transparent*, *and funding is in the hands of a few*; and *wasted spending of research funding*. We identified 10 larger themes that encompass the individual challenges identified by respondents that exist in China’s research environment: (1) promotion of instant-success/short-term outlook; (2) research funding; (3) excessive governmental/administrative/bureaucratic intervention; (4) evaluation/assessment system; (5) reliance on human relations; (6) lack of support for research faculty; (7) academic misconduct/scientific integrity; (8) structured uncertainty/impetuosity; (9) education system; and (10) reward/incentive system. Additionally, we included an eleventh and twelfth theme—an *Other* category to capture the responses that did not fit into one of the ten themes, and a *None* category to capture the responses of individuals who stated that there were no challenges to China’s research environment. For each of the twelve themes, we calculated the total number of individuals and percentage of individuals that identified at least one challenge within each theme. Cochran’s Q-test was used to assess if there were significant differences in the percentage of individuals who identified at least one challenge within each larger challenge theme. Pairwise comparisons were then used to assess how the twelve challenge themes differed significantly from one another.

We also analyzed the responses to the free-response question “*What changes (if any) do you think would improve the research environment in China*?” A total of 448 responses were received. Five were discarded because the content did not address the question of interest or the answer was unclear. We did not want to make any assumptions on the meaning of the responses and chose to remove them from our analyses. A total of 443 responses were used in our analyses. Following the same protocol as those used to assess challenges to China’s research environment, we translated all responses to English, identified the unique solutions respondents suggested as ways to improve China’s research environment, and coded all responses according to each solution (i.e., 0 if a solution was not suggested in the response, and 1 if the solution was suggested in the response). Thirty-nine unique suggestions were identified as potential ways for China’s research environment to be improved. These unique suggestions were grouped into 10 larger themes: (1) decrease the amount of governmental/administrative/bureaucratic intervention; (2) change the evaluation/assessment system; (3) change the research funding process/system; (4) increase faculty support/provide better treatment of faculty; (5) learn from foreign/international institutions; (6) increase research funding investment; (7) increase academic integrity/encourage professionalism; (8) enact political/social reforms; (9) other; and (10) no way to change/I don’t know. The tenth category is one in which individuals indicated that there are no possible solutions to improve China’s research environment or they are unsure of how to improve China’s research environment. For each of the ten themes, the total number of individuals and percentage of individuals that identified at least one solution within each theme were calculated. Cochran’s Q-test was used to assess if there were significant differences in the percentage of individuals who suggested at least one solution within each larger theme. Pairwise comparisons were then used to assess how the distinct themes of solutions differed significantly from one another.

## Results

### Foreign degree holders (returnees) in comparison with domestic degree holders

Of the 731 survey respondents, 700 (96%) received PhDs, 23 (3.1%) received Master’s degrees, 5 (0.68%) received Bachelor’s degrees, and 3 (0.41%) received another type of degree as their terminal degree. One hundred and twenty-two respondents (17%) received their terminal degrees from abroad and 609 (83%) received their terminal degrees from Chinese institutions of higher education. Among those who received foreign degrees, 46 (38%) studied in the United States, 24 (20%) studied in Japan, 9 (7.4%) studied in Germany, and 8 (6.6%) studied in England. Of those who received a PhD from abroad, 82% remained in the host country to work while 18% returned immediately to China upon graduation. Moreover, 19% currently hold a faculty position in both China and a foreign institution.

**Fig 1:**
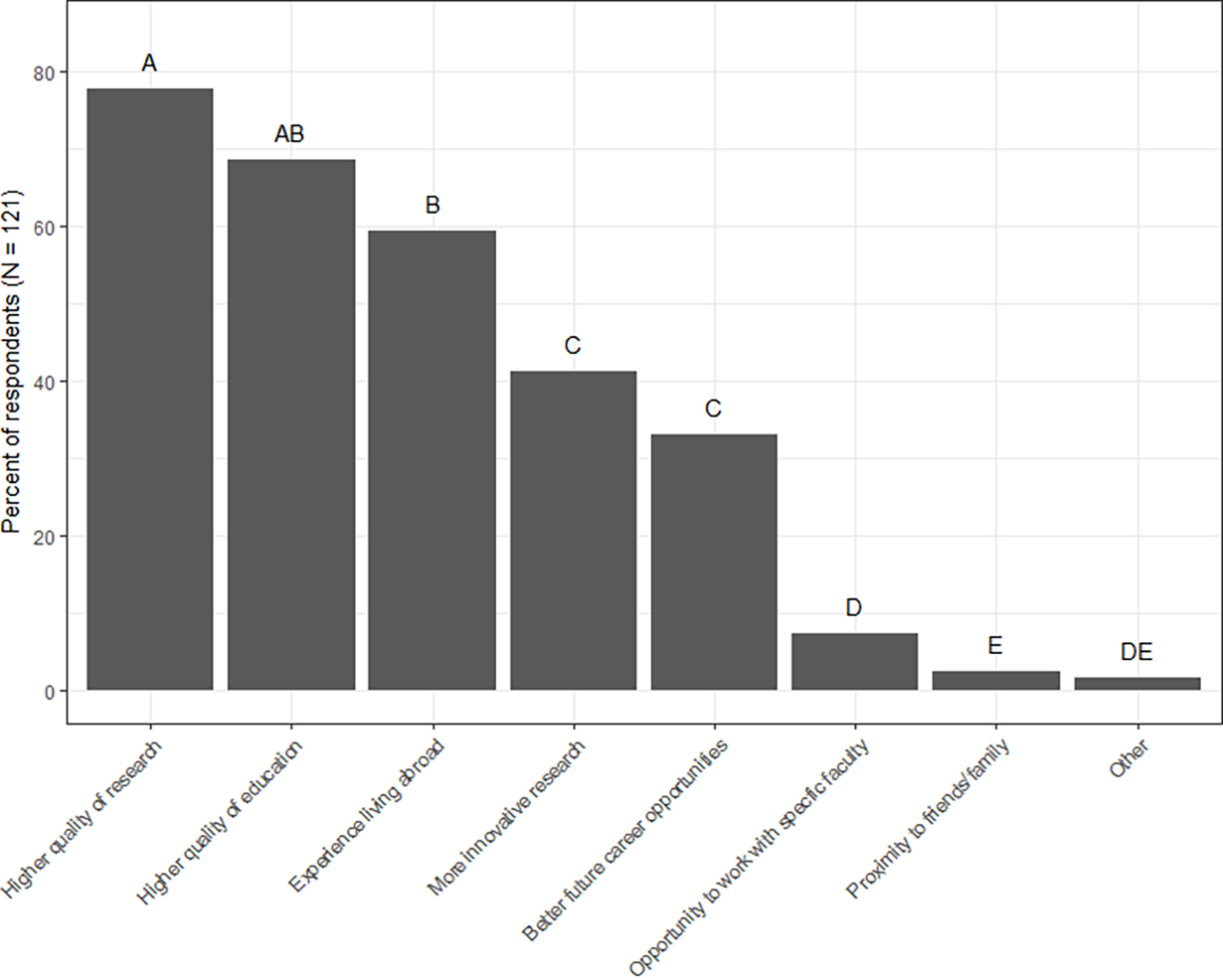
Reasons for studying abroad. Percent of returnees that selected each factor as a reason behind their decision to study abroad (N = 121). Respondents were allowed to select multiple factors. Pairwise comparisons using the Wilcoxon sign test were performed post-hoc to determine how factors differed significantly from one another. Letters represent significant differences in percent of respondents at the α = 0.05 level.

Individuals who studied abroad indicated that they chose to do so because it provided them with the opportunity to do *higher quality research in their field* (78%); have a *higher quality of education* (69%); *experience living abroad* (60%); have the *ability to do more innovative research* (41%); and have *better future career opportunities* (33%) (Fig 1). Note that percentages do not add up to 100% because respondents were allowed to select all reasons that applied for why they chose to study abroad. *Opportunity to work with specific faculty*, *proximity to friends/family*, and *other* reasons were selected by less than 10% of respondents. Results from Cochran’s Q-test indicated that reasons for studying abroad fell into five significantly different groups with *higher quality of research* and *higher quality of education* as the most selected reasons for why individuals studied abroad.

**Fig 2:**
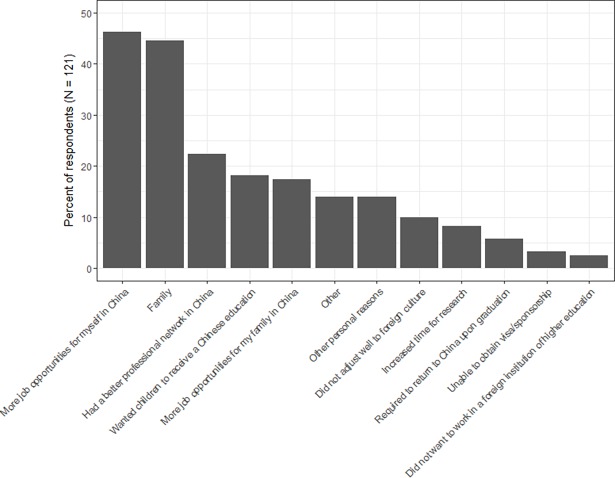
Reasons for returning to China. Percent of returnees that selected each factor as a reason for returning to China after completing their studies abroad (N = 121). Respondents were allowed to select multiple factors.

Returnees also indicated that the top two reasons for returning to China were *more job opportunities [for themselves] in China* (46%) and *family* (45%). This was followed by *having a better professional network in China* (22%); *wanting their children to receive a Chinese education* (18%); *more job opportunities for their families in China* (17%); *other* reasons than the ones listed in the survey (14%); and *other personal reasons* (14%) (Fig 2). The following reasons for returning to China were selected by less than 10% of individuals who studied abroad: *did not adjust well to foreign culture* (9.9%); *increased time for research* in China (8.3%); *required to return to China upon graduation* (5.8%); *unable to obtain a visa/sponsorship* to stay in host country (3.3%); and *did not want to work in a foreign institution of higher education* (2.5%). No respondents selected *fewer administrative responsibilities at Chinese institutions* as a reason for returning.

**Fig 3:**
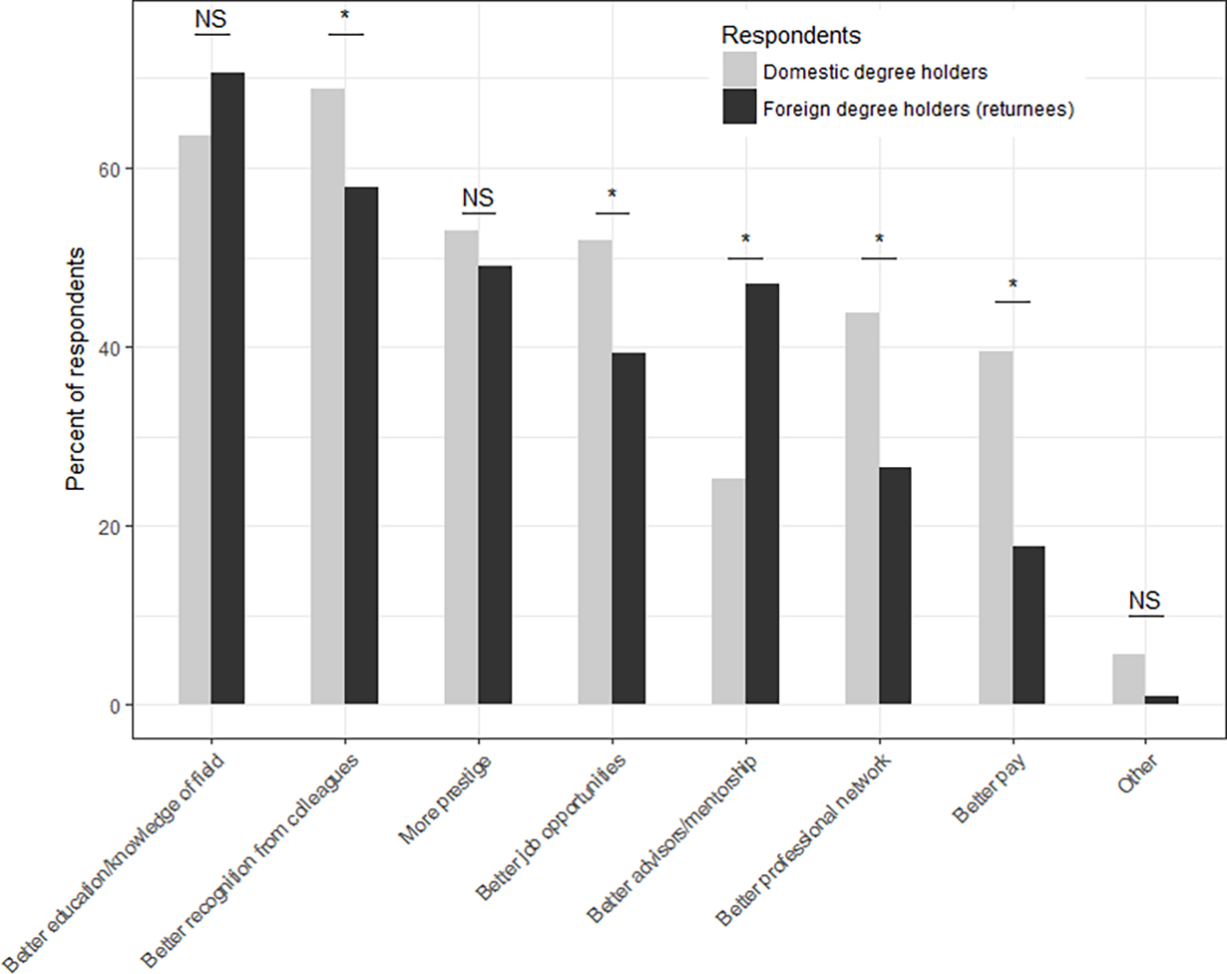
Perceived advantages to having a foreign degree. Percent of respondents by type of degree (domestic vs. foreign) that selected each factor as a perceived advantage to having a foreign degree. Two-sample proportion tests were used to evaluate if the percent of respondents who perceived an advantage differed between those who were domestic degree holders (i.e., graduated from Chinese institutions of higher education) and those who were foreign degree holders (i.e., returnees). NS indicates no significant difference; and * indicates significant difference between the percent of degree and foreign degree holders at the α = 0.05 level.

Among individuals who received their terminal degrees from abroad and who do not hold dual positions in China and abroad (N = 96), 60% returned to China within 5 years; 19% returned after 5 to 7 years; 10% returned after 8 to 10 years; and 10% returned after 10 years or more abroad. Among those who received a foreign degree, 84% felt that their foreign degree provided them with advantages, while 16% did not feel that they received any advantages because of their foreign degree. Among those who felt they have received advantages because of their foreign degree (N = 102), 71% indicated that they received *better education/knowledge of their field*; 58% indicated that they received *better recognition from colleagues in China once they returned*; 49% indicated that they received *prestige*; 47% indicated that they had *better advisors/mentorship*; 39% indicated that they had *better job opportunities* compared to what they would have obtained if they received their terminal degrees from a Chinese institution of higher education (Fig 3). A smaller percentage of individuals indicated that a foreign degree provided them with a *better professional network* (26%) and *better pay* (18%).

Similarly, 82% of those who received a PhD from a Chinese institution of higher education also indicated that they believed there are advantages to having a foreign degree over a domestic degree. They perceived *better recognition from colleagues in China once foreign degree holders returned* as the biggest advantage of having a foreign degree (69%); followed by *better education/knowledge of one’s field* (64%); *prestige* (53%); *better job opportunities* (52%); *better professional network* (44%); *better pay* (40%); and *better advisors/mentorship* (25%) (Fig 3). Two-sample proportion tests indicated that there were no differences between the proportion of foreign and domestic degree holders who felt that foreign degrees offered more *prestige* (P = 0.53) and a *better education/knowledge of their field* (P = 0.22). A significantly higher percentage of domestic degree holders, however, felt that foreign degrees provided *better recognition from colleagues in China* (P = 0.04); *better professional networks* (P < 0.01); *better job opportunities* (P = 0.03); and *better pay* (P < 0.001). Foreign degree holders, on the other hand, were significantly more likely to indicate that a foreign degree provided them with *better advisors/mentorship* (P < 0.001).

### Satisfaction measurements

A majority of respondents (55%) indicated they were either *satisfied* or *very satisfied* with their current position. A smaller percentage (32%) indicated that they were neither *satisfied* nor *dissatisfied* with their current position, and 13% of individuals indicated that they were *dissatisfied* or *very dissatisfied* with their current position. A two-sample proportion test showed that the proportion of respondents who were *satisfied* or *very satisfied* was significantly higher than the proportion who were *dissatisfied* or *very dissatisfied* (P < 0.001).

The proportion of respondents who were *satisfied* or *very satisfied* with the research culture in their home departments (34%) did not differ significantly from the proportion who were *dissatisfied* or *very dissatisfied* (30%) (P = 0.14). Thirty-six percent of respondents indicated they were neither *satisfied* nor *dissatisfied* with the research culture in their home departments.

With regards to satisfaction regarding the overall research culture in their respective fields, the proportion of individuals who were *satisfied* or *very satisfied* (33%) was significantly higher than the proportion who were *dissatisfied* or *very dissatisfied* (27%) (P = 0.01). Thirty-nine percent of respondents indicated they were neither *satisfied* nor *dissatisfied* with the overall research culture in their fields.

With regards to satisfaction with the overall research culture in China, the proportion of respondents who were *dissatisfied* or *very dissatisfied* (42%) was significantly higher than the proportion of those who were *satisfied* or *very satisfied* (19%). Thirty-nine percent of respondents indicated they were neither *satisfied* nor *dissatisfied* with the overall research culture in China.

### Publication incentives

Of the 731 survey respondents, 550 (75%) indicated that their department or university offer incentives to research faculty who publish in an English-based foreign journal. Of the 550 individuals who indicated incentives are provided, 412 provided detailed descriptions of the kinds of incentives offered by their home departments or universities. Publication incentives were all cash rewards but the amount rewarded varied widely by departments and universities. Forty-two percent of respondents indicated that the amount received was based on the journal’s ranking. For example, three professors from three different departments at Beijing Normal University noted the incentives they would receive: 5,000 RMB for each SCI publication (geography department); 1,000 RMB for each SCI and EI publication (information science and technology department); and no rewards are given for average articles, rewards are reserved for high quality articles (environmental science department).

On average, incentives ranged from 500 to 5,000 RMB for a SCI or EI publication. Respondents noted that cash incentives are typically reserved for whoever holds the first-author position; are only awarded if the impact factor is above a certain number; and are generally tiered with higher incentives provided for publications in high-ranked journals, less for middle-ranked journals, and little to no cash incentives for low-ranked journals. Many noted that for publications in Cell, Nature, or Science, the incentives were often much higher, ranging from tens of thousands of RMB to several million RMB. A respondent from Huazhong University of Science and Technology stated the following:

For IF>30, the reward is 100,000RMB; for 10<IF<30, the reward is 30,000 RMB. If your paper enters the Essential Science Indicators (ESA) after a year of publication, then you get an 80,000 RMB bonus. A [publication in a] level-one journal ranked by the Chinese Academy of Sciences [receives a] 20,000 RMB reward; a level-two journal [publication] receives 5000 RMB.

A respondent from Shanghai Jiaotong University echoed that:

For Science and Nature [publications], there are special incentives ranging from 50,000–100,000 RMB. For top-ranked journals within a discipline, rewards are around 5,000–10,000 RMB. There are no incentives or rewards for publishing in regular SCI journals.

### Challenges to China’s research environment

**Fig 4:**
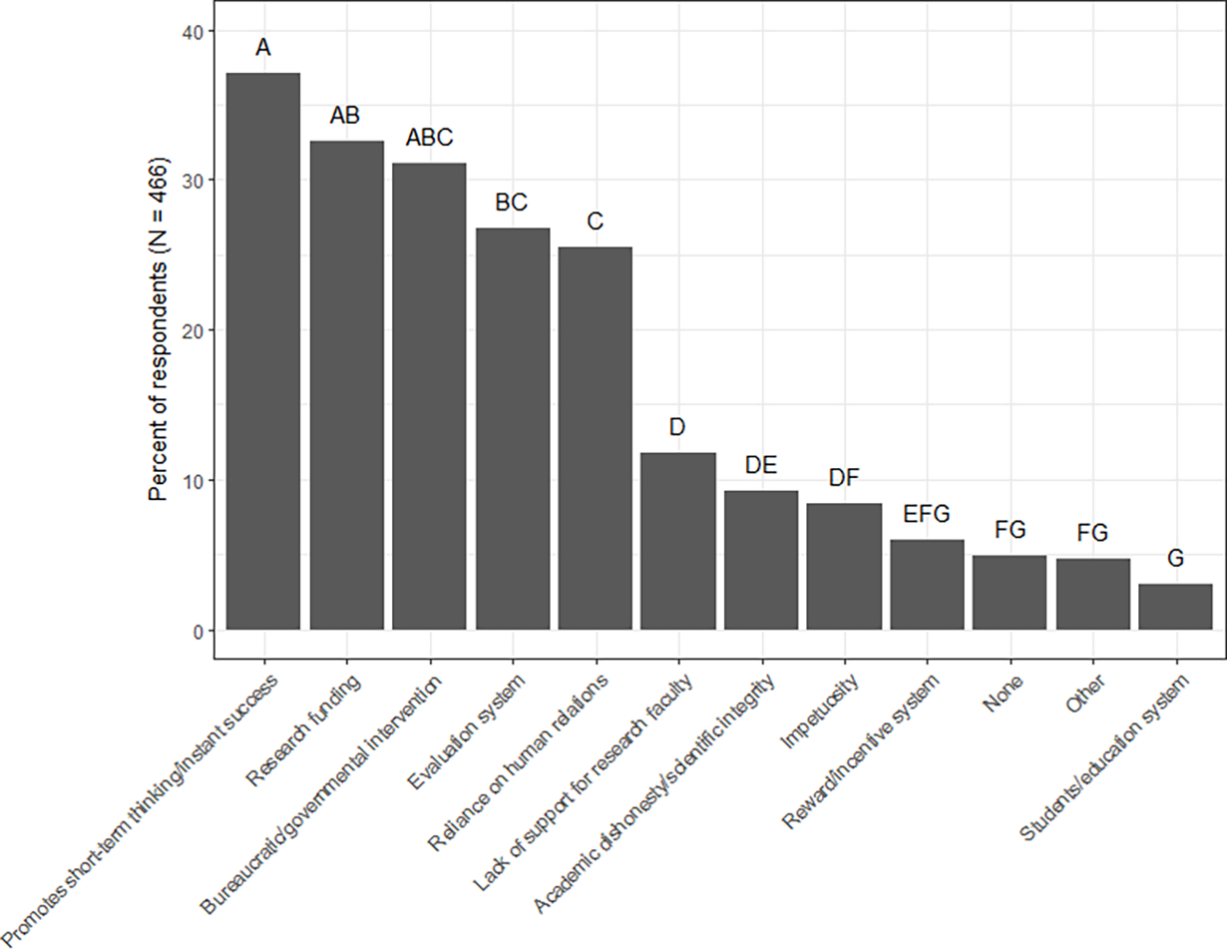
Challenges to China’s research environment. Percent of respondents that indicated the challenges that are facing China’s current research environment (N = 466). Pairwise comparisons using the Wilcoxon sign test were performed post-hoc to determine how challenges differed significantly from one another. Letters represent significant differences in percent of respondents at the α = 0.05 level.

Text analysis of the 466 free responses indicated that the biggest challenge to China’s current research environment is that it *promotes short-term thinking and instant success* (37% of all respondents). This was followed by *research funding* (33%); *too much bureaucratic or governmental intervention* (31%); the *evaluation system* (27%); a *reliance on human relations* (i.e., *guanxi*) (26%); a *lack of support for research faculty* (12%); *academic dishonesty/scientific integrity* (9%); a system marked by *impetuosity* (8%); the *reward/incentive system*; and *students and the education system* (3%) (Fig 4). Five percent of respondents indicated that they did not feel there were any challenges to China’s current research environment and 5% indicated other challenges that did not fall into one of the larger twelve themes. Results from Cochran’s Q-test showed that the 12 challenge themes fell into 7 statistically different groups. The top three challenges (*promotes short-term thinking and instant success*, *research funding*, and *too much bureaucratic or governmental intervention*) fell into one statistically significant group. Detailed descriptions of the top five challenges follow.

#### Challenge 1: Short-term thinking and instant success

Respondents stated that China’s current research environment does not promote innovative or original research because quantity (e.g., number of publications) is emphasized over quality of research. The environment encourages researchers to engage in research activities that can lead to short-term, instant success such as going after low-hanging fruit and producing a quick turn-around publication rather than engaging in long-term, innovative, and more risky research that may or may not lead to a scientific breakthrough, and may take decades for results to be realized.

A respondent from Shanghai Jiaotong University wrote that “the pursuit of the number of publications is a short-sighted behavior; nobody is willing to do long-term research.” A respondent from Sichuan University stated that “there is not enough freedom to pursue original, innovative research” and that the “reward system promotes short-term research.” A respondent from the South China University of Technology noted that “there is no ‘bigger picture’ in research projects, there is not complete independence and freedom to do research.” A respondent from Sun Yat-Sen University argued that:

People do research because they are forced to do research, people publish publications because they have to publish publications. There is too much focus on instant success. Very few people are actually interested in doing research, they only do it because they are forced to. Real research that addresses any [real] research problems or is of actual quality is very low.

#### Challenge 2: Research funding

Concerns about research funding is comprised of three sub-components: research funding is too low; research funding is unfair, not transparent, and received by too few people; and resources are wasted or are unreasonably spent. Of the 152 respondents who indicated that research funding posed a challenge to China’s research environment, 89% noted that the funding system was unfair, not transparent, and monopolized by a select few. Respondents noted that one’s *guanxi* (i.e., connections) still plays a role in determining one’s research funding, and that funding is often restricted to a select few belonging to certain ‘research circles.’ A respondent from Huazhong University of Science and Technology succinctly stated that “research monopolies get all the research funding. General researchers find it very difficult to obtain funding. To get research funding, it is primarily based on human connections and *guanxi*, and not on an individual’s research abilities”.

#### Challenge 3: Too much bureaucratic or governmental intervention

Excessive governmental intervention in directing scientific research was the second biggest challenge identified by respondents. Individuals indicated that the system is too rigid and lacks academic freedom; that there is strong pressure for researchers to follow official ideologies and standards; and that administrators and bureaucrats have too much power, that power is only in the hands of a few, and that oftentimes, those in power have little to no expertise in what they oversee. An example of governmental overreach noted by several respondents is that the central government regulates the number of Ph.D. students a university is allowed to accept each year rather than the university itself. This was viewed as an unnecessary and obtrusive interference by the central government on a minor matter that should be regulated by universities. One respondent (university not indicated) noted that the “government has way too much intervention in higher education [and] does not rely on the natural progress of higher education to govern.” Graduate student recruitment may seem like a relatively minor issue, but a respondent from Nankai University indicated that it can have far-reaching consequences by noting:

Recruitment of graduate students is very inflexible. Many professors have projects but are unable to get graduate students. One reason is related to national-employment statistics, another reason is the management agency for graduate enrollment is too tightly controlled. Frequently, we're not allowed to get PhD students, this has caused a lot of early and mid-career faculty researchers to halt their research and not be able to complete their research projects.

A respondent from Central South University stated that “there is too much government involvement, scientists and researchers’ opinions are not respected or valued.” Another respondent noted that “government intervention is on the high side; the vast majority of things are government controlled or directed.” These sentiments were echoed by many respondents. A number of respondents noted that academic freedom remains an issue to China’s research environment and culture. A respondent from Sun Yat-Sen University wrote that:

There is still not enough academic freedom in higher education. If the Central Government makes one statement, even if it is not fair, all of the universities have to follow suit. The university president does not represent the school, [s(he)] only responds to higher ups [i.e., government officials].

A respondent from Shanghai Jiaotong University remarked that “[academic] policy reforms only apply for ordinary professors, administrators and those up top are not affected by policy changes at all. Very unfair.” A respondent from Peking University stated that

The biggest problem [in China] is that the goal of research is not to prove/disprove scientific theories nor is it to solve societal problems. The research goal in China is for the researcher to get better academic status and for personal interest through doing research. The damage to academic freedom right now is not because of political factors but mainstream academic influence and excessive academic competition.

#### Challenge 4: Evaluation system

Unlike the other top challenges, *evaluation system* is not composed of smaller components that make up this challenge. Problems associated with the evaluation system were so numerous that we designated it as its own challenge theme. Respondents were also the most verbose about the evaluation system, sometimes providing a paragraph description on the topic while simply listing other challenges. Responses indicated that the evaluation system hinders innovative research; is unfair; is too focused on quantitative measurements of research; places a high level of administrative burden on researchers; does not effectively measure research quality; and promotes short-term outlook. Depending on their home institution, researchers can be evaluated on a monthly, bi-annual, and/or annual basis on their research and teaching performance. Generally, research performance is evaluated by the number of first author or corresponding author publications published, the journals in which the publications were published, journal impact factors, amount of funding received and the like. Teaching performance is typically evaluated by the number of courses taught, number of Master’s and/or PhD-level students mentored, number of graduating Master’s and/or PhD-level students, etc. Researchers are strongly recommended to achieve research and teaching ‘milestones’ by evaluation time to avoid financial or other punitive sanctions.

The evaluation system, problematic in and of itself, has also contributed to other challenges in China’s research environment. As noted by a respondent from Fudan University:

The amount of funding one receives is a very large indicator in one’s performance evaluation; people fabricate or plagiarize papers so that they can pass their annual performance evaluations. The same is true for title [i.e., academic rank] evaluations.

The sentiment that publication requirements induce plagiarism, fabrication of results, and academic misconduct in general was echoed by many respondents. Another respondent from Fudan University stated that the evaluation system “only considers the amount of funding received and the [journal] ranking of where articles are published, [it] does not consider the true quality and impact of the research being done, the value of the research, or the social impact of the research.” Respondents also noted that there are too many evaluation assessment indicators; that researchers can get too overwhelmed by the evaluation system; and that researchers sometime focus more on passing their evaluations than they do on research. The overall impact of the university evaluation system on China’s research environment can be summarized from the response of a respondent at Nanjing University:

Because the evaluation system is fast paced, it forces researchers to conduct short-term research to meet all of the requirements such as meeting their annual quota on total number of publications published. The environment facilitates the evaluation and assessment system, but it is not conducive to the cultivation of scientists.

#### Challenge 5: Reliance on human relations

As noted in *challenge 2*: *research funding*, relying on one’s connections to get ahead is still a common feature in China’s research environment. This has led a phenomenon called *kao shantou*, literally translated to “rely on the mountain top.” This is when researchers organize into groups with the understanding that individuals help and protect those within the group. For example, when an individual within the group gets ahead or is promoted to a position of power, (s)he show favoritism only to those in his/her group and vice versa. The ‘mountain top’ is the individual who is in a position of power and on whom everyone else in the group relies.

### Suggestions on how to improve China’s research environment

**Fig 5:**
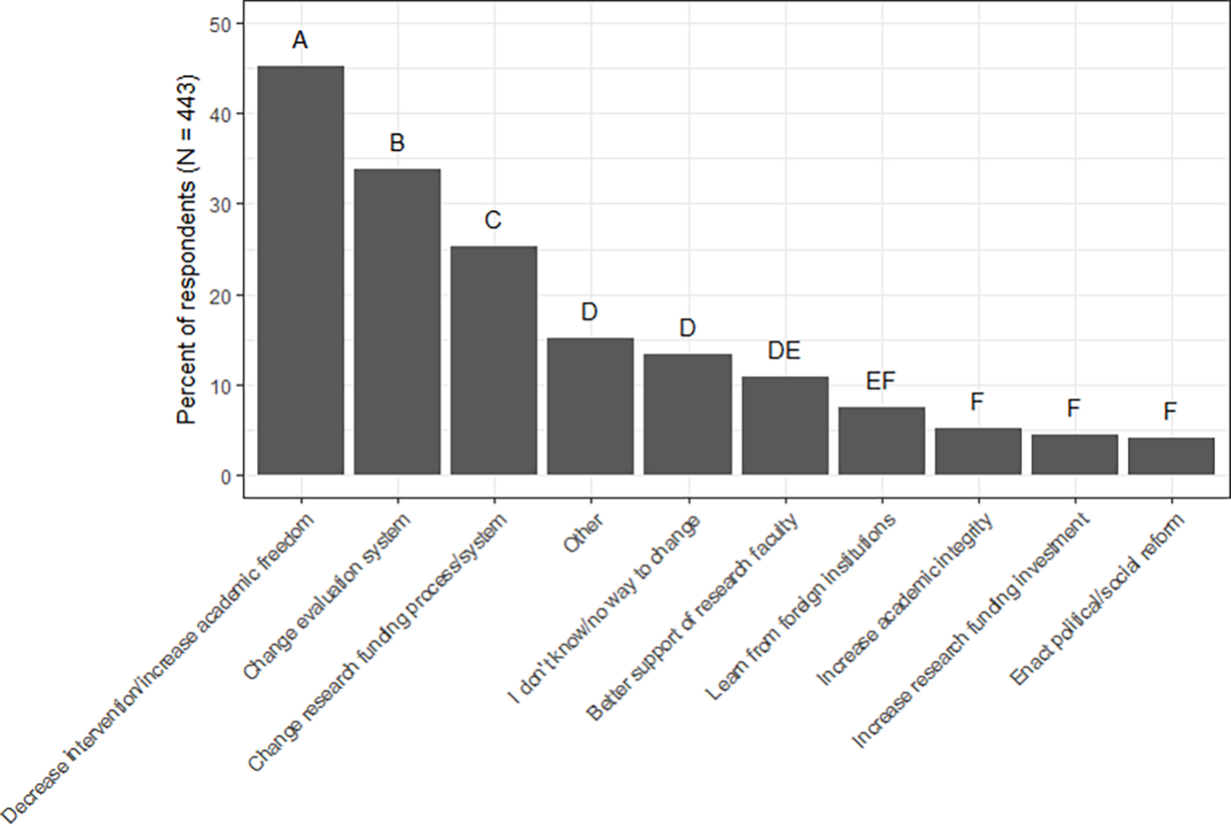
Suggestions on how to improve China’s research environment. Percent of respondents that provided suggestions on how to improve China’s current research environment (N = 443). Pairwise comparisons using the Wilcoxon sign test were performed post-hoc to determine how recommended solutions differed significantly from one another. Letters represent significant differences in percent of respondents at the α = 0.05 level.

Text analysis of the 443 free responses showed that 45% of respondents believed that decreasing governmental, administrative, and bureaucratic intervention, and increasing overall freedom in higher education would improve China’s research environment. This was followed by making changes to the evaluation system (34%); making changes to the research funding process (25%); other changes (15%); no way to change/I don’t know (13%); increase support and treatment of the research faculty (11%); learn from foreign or international research environments (7%); increase academic integrity, professionalism, and civility, and punish academic fraud (5%); increase research investment (4%); and have political and social reforms (4%) (Fig 5). Results from Cochran’s Q-test showed that suggestions on how to improve China’s research environment fell into 6 significantly different groups. The top three suggestions fell into three statistically significant groups; detailed descriptions of the top three suggestions follow.

#### Decrease intervention and increase freedom

Decreasing governmental, administrative, and bureaucratic intervention, and increasing overall freedom in higher education consists of 9 individual sub-components. Respondents suggested that the government decrease the number of established research projects and allow researchers to apply for more self-directed research; limit its role in higher education and give the academic community more control; establish a fair, open, and transparent system; give researchers more academic freedom and freedom to explore research topics; gets rid of its official line of thought; let research be dictated by market forces and increased privatization; establish a more fair reward system or abolish the reward system altogether; put an end to talent programs; and relax graduate student enrollment requirements. A respondent from Huazhong University of Science and Technology provided a succinct summarization of how to improve China’s research environment:

Give professors a lot more freedom. Do not use all sorts of restrictions and regulations…to manage professors. In regards to the amount of research funding and the number of publications, I would recommend decreasing both. … I suggest that a lot of the funding process get rid of age requirements or to loosen the age restrictions. I recommend that people have the freedom to apply to all programs, have fair competition. Also, research cannot be so short-term in scope. Requiring a set number of publications per year, how can this result in research that has any real impact. [With regards to research] funding in China, I recommend that it increases in transparency, and becomes decentralized. In regards to undergraduate education, I also recommend that it is the quality that counts, not quantity. Reduce the number of obsolete or low quality undergraduate courses. Curriculum design should follow that of foreign institutions.

#### Change the evaluation system

Respondents recommended that the research system allow room for failure; to stop focusing on instant success; to increase long-term and basic research; to get rid of quantitative indicators; and to restructure the evaluation system. Respondents recommended a number of ways to improve the evaluation system but no suggestion was recommended by a majority of respondents. Recommendations included emulating foreign institutions; changing to a peer review system; allowing individuals to choose to focus solely on teaching or solely on research; establishing an independent, third-party evaluation system and advisory body. There was, however, consensus among respondents that the evaluation system should be fair and transparent, should have less reliance on quantitative indicators, and should emphasize long-term or basic research over short-term success.

#### Change the research funding process

Respondents proposed that the research funding process be open and fair. To do so, respondents recommended that human interactions be limited in the allocation of resources; the formation of research circles should be avoided or minimized; and funding should be based on a peer review system or some form of anonymous, double-blind, or third-party review system to avoid human bias.

## Discussion

### Returnees and homegrown scholars

We found that 96% of our survey respondents have doctorate degrees and 3% have Master’s degrees. This is a remarkable increase even from the early 2000s, when it was estimated that only 30% of all faculty members held postgraduate degrees, a consequence of China’s Cultural Revolution [43]. This increase is expected as the first- and second-generation of professors after the Cultural Revolution retire and are replaced by those with doctorate degrees. We recognize that the overall percentage of Chinese faculty members with postgraduate degrees is likely lower than what we observed for two reasons: (1) our survey respondents come from China’s top-tiered institutions and it is reasonable to assume that China’s second- and third-tier institutions may not have as stringent requirements for their faculty members; and (2) as this survey was distributed electronically, we acknowledge that older faculty members (i.e., those that are most likely to not have a postgraduate degree) may have been less likely to respond thereby skewing our respondents to those with postgraduate degrees.

We also found that the vast majority (83%) of our survey respondents received their terminal degree from a Chinese institution of higher education and that less than one-fifth hold a foreign degree. We note that the overall percentage of faculty members who hold foreign degrees is likely lower, since those who return to China preferentially select top-tiered institutions since they have better resources and can offer more competitive benefits packages to attract those who have studied abroad. The percent of foreign-trained Chinese faculty members, however, is likely to increase over time. Returnees identified *more job opportunities* and *family* as the top two reasons for returning to China. Combined with competitive salaries, and start-up and benefits packages offered by China’s incentive programs (e.g., Thousand Talents Program or Young Thousand Talents Program), increased numbers of foreign-trained Chinese researchers may find returning to China a more attractive option than those in the past. Whether more returnees will be able to bring about changes to China’s research environment, however, is a different question altogether. Given that the top four challenges identified by survey respondents are top-down in nature and the fifth challenge (the reliance on human relations) is a deeply ingrained cultural phenomenon, changing China’s higher education research environment will likely require more than just increased numbers of returnees. Just like the challenges, which are predominantly top-down, changing China’s research environment will likely require a top-down approach as well. Until China’s central government cedes control back to the universities and academic community, faculty members may have little to no sway in implementing changes to the research environment.

Survey results also showed that approximately four-fifths of both returnees and home-grown scholars acknowledged that having a foreign degree provides an individual with advantages in China. The two groups differ, however, on the types of advantages that are provided. Both agreed that a foreign degree provides individuals with a better education and/or better knowledge of their field, and that it provides individuals with more prestige. Homegrown scholars, however, were statistically more likely to attribute job-related and financial success to having a foreign degree than returnees. Homegrown scholars believed that a foreign degree provided a person with better pay, better job opportunities, better professional networks, and better recognition from colleagues. Returnees, on the other hand, were more likely than homegrown scholars to believe a foreign degree provided them with better advisors/mentorship.

Differences in perceived advantages between returnees and homegrown scholars may contribute to animosity between the two groups. Past studies [10, 44–46] have alluded to the possibility that homegrown scholars may be resentful of the advantages that returnees receive, particularly through repatriation programs such as the Thousand Talents Program [10]. This study is the first, to our knowledge, to show that both homegrown scholars and returnees overwhelmingly acknowledge that there are numerous advantages to having a foreign degree, and that homegrown scholars feel that returnees have greater work and financial success because they have foreign degrees. It is important to note that none of our survey respondents indicated that there was any animosity, resentment, or jealousy between the two groups. Many homegrown scholars have stated that returnees are important to improving China’s higher education and research environments [10]. We only note that by providing returnees with extra benefits (e.g., more pay, better start-up package, housing allowances or subsidies, etc.), the Chinese central government creates an unfair environment that may hinder collegiality and collaboration. Similarly, by providing advantages and bestowing titles on returnees for which homegrown scholars are ineligible, the government tacitly diminishes those who were educated by Chinese institutions of higher education by implicitly sending a message that Chinese institutions of higher education are not as good or as valued as foreign institutions.

### Challenges and opportunities

Of the 466 respondents who identified challenges in China’s research environment, only 5% stated that there were no challenges. Respondents described numerous challenges, which we categorized into broader challenges for reporting ease and to better account for interdependencies among challenges. Despite being presented as separate challenges, we note that all challenges are interconnected and contribute to one another. For instance, even though *short-term thinking and instant success* was the most identified challenge, *decreasing governmental*, *administrative*, *and bureaucratic intervention*, *and increasing overall freedom* was the most suggested solution on how to improve China’s research environment. Overall, China’s research environment is hindered by a central government that insists on regulating both the research and academic aspects of the system to fast-track China’s development and international standing. Instead of allowing research to direct itself based on findings and progress, the central government identifies a national research priority list that has specific amounts of funding allocated to each research priority. Oftentimes, there are also regional and local research priority lists. To get funding, researchers must align themselves with these research priorities or restructure their research to fall in line with these priorities. Respondents asked for increased freedom in determining their own research trajectory or research project. Specifically, respondents noted that they would like to see more non-designated research funding allowing researchers to pitch their own ideas rather than responding to a targeted research request.

Similarly, respondents were discontent with the amount of government overreach in academia. The most brought up example is that the central government dictates the number of graduate students a university is allowed to enroll each year. The university then allocates a specific number of graduate students to each department depending on how highly it values that research field. A department that specializes in a research area that is targeted by the national research priority list will typically receive more enrollment slots than one that does not appear on the list. Respondents noted that because of this allocation, each professor can typically enroll only one graduate student a year, regardless if he/she has the funding to support more. This also results in situations in which professors are unable to pick which graduate students they would like to accept, since sometimes they only have one applicant who they must accept if they hope to have any graduate students to help them with their research. The overall sentiment among respondents is that universities should be given the right to govern themselves, and academic affairs should be solved by those in the academic community–and not by individuals who have little expertise with academia but happen to be in positions of power.

In China’s urgency to become a key player in the scientific community, the government has focused on research quantity over research quality. This has resulted in the much maligned evaluation system that respondents are keen to see changed. As many respondents indicated, more faculty members are spending their time trying to meet their university and department requirements for research and teaching than actually doing research and teaching. Many respondents echoed that this has resulted in a situation where very few still enjoy doing research. As stated before, the goal of research in China is no longer seen as about the pursuit of knowledge; rather, it has become a pursuit designed to meet quantitative indicators for one’s evaluation.

The current evaluation system is a clear source of distress among survey respondents, one that, many argued, does not adequately assess an individual’s research potential or quality. This has resulted in an environment where researchers are forced to engage in short-term, low-hanging research to meet publication requirements. It has, as respondents noted, resulted in a proliferation of academic misconduct and fraud that has marred China’s scientific reputation [47–50]. The latest case that resulted in a retraction of 107 papers by 521 Chinese authors resulted in an investigation by the Ministry of Science and Technology [51]. Investigators concluded that authors and third party agencies provided phony reviewers and compromised the integrity of the peer review system. After each widely acknowledged incident and public outcry of plagiarism, fraud, or buying of co-authorship, the government has pledged to change the system to avoid future cases of scientific misconduct. Despite its promises, very little has changed. The current evaluation system, as criticized by respondents, contributes to increased scientific misconduct by Chinese researchers and does not promote innovative or quality research. Failure is a natural and essential part of science. Without it, progress cannot be made. China’s current evaluation system does not allow room for failure, it does not even tolerate it.

Another clear source of frustration among survey respondents is the connection between *guanxi* and research funding. Since funding is a common part of a researcher’s evaluation, it is understandably a source of stress. This is exacerbated by the fact that funding appears to be an unfair process in which select individuals receive most of the funding and that one’s *guanxi* plays a large role in determining if one gets funding. The role of *guanxi* in China has been well documented (see [52–55]) and we do not expand upon it in this study. We note that *guanxi* in academia and research appears to have transitioned itself to a new phenomenon—this idea of a research circle where those within the circle look out for each other. It appears that those within the research circle benefit from better access to funding and those outside the circle may have trouble getting adequately funded.

As evidenced from the results section, respondents provided a myriad of ways to improve China’s research environment. It seems clear that despite all of the challenges facing China’s research environment, the vast majority of respondents are hopeful that the environment will improve in the future. Although many solutions were offered, it seemed clear that *decreasing governmental*, *administrative*, *and bureaucratic intervention*, *and increasing overall freedom* would probably result in the biggest improvement to China’s research environment. Respondents indicated that they would like to see more power and control be returned to universities and the academic community in general. China has made tremendous progress over the past three decades and is now entering an era where the government should take on more of an oversight body role and return control to the academic and research communities.

We acknowledge that many of the challenges identified by survey respondents are not specific to China’s research environment. The pressure on faculty members to “publish or perish,” and the use of quantitative metrics, such as number of publications and amount of funding, to make decisions on promotions within the academic system are also challenges faced by faculty members within the U.S. [56–59]. There may be many similarities between China’s research environment and that of the U.S. but systematic studies are needed on both sides to conduct comparative analyses. The trajectory of China’s research environment is likely to differ from that of the U.S. given cultural and political differences but that does not mean that the two countries cannot learn from one another on ways to improve their respective research environments.

## Conclusions

China’s hope of becoming a global innovator has roots that predate the Xi Jinping era (for a detailed historical discussion, see [60]). In 2006, President Hu Jintao launched the 15 year Medium and Long-Term Plan for S&T Development, which called for China to embark on an ambitious program of “indigenous innovation” [61]. In 2013, under President Xi, the “Made in China 2025” (中国制造2025) initiative was announced, calling for innovation-driven, high-quality manufacturing, nurtured by investment in human talent. Ten priority sectors were identified, most of which will require innovative technologies to become globally competitive. Two years later, China’s 13^th^ Five Year Plan (2016–2020) was announced, which included specific metrics for achieving innovation goals in cutting-edge fields such as nanotechnology and quantum communication; specific numbers of scholarly citations, patents, and technical contracts; and even “percentage of citizens with scientific literacy” [62]. The plan emphasized “talent development” as a priority strategy [62], calling for strengthening “the training and use of innovative scientific and technological talents from minority groups, and value and increase the proportion of female scientific and technological talents” [62]. Such goals are to be achieved through both educational reform and drawing on foreign talent [62]. The goal of “comprehensively increasing the innovation capacity of universities” is to be achieved in number of ways, including “accelerate the construction of a modern university system with Chinese characteristics, implement and enlarge the legal autonomy of institutions of higher learning, promote education innovation” [62].

At his three-hour speech to the 19^th^ Communist Party Congress on 18, 2017, President Xi Jinping reaffirmed his “Chinese dream:” China, he stated, “is approaching the center of the world stage” and will become “a global leader in terms of comprehensive national strength and international influence.” By 2050, he said, China will become “a global leader in terms of comprehensive national strength and international influence approaching the center of the world stage” [63]. Innovation in science, technology, and resulting goods and services is central to this vision but a modern university system with Chinese characteristics will likely face the challenges identified in this paper.

## Supporting information

S1 Appendix Chinese university rankings by WuShulian and China Alumni Network. (DOCX)

S2 Appendix Survey introductory letter in Mandarin Chinese and English. (DOC)

S3 Appendix Survey in Mandarin Chinese. (DOCX)

S4 Appendix Survey in English. (DOC)

S5 Appendix Calculations and results for survey non-response bias. (DOCX)
