# Supplementary material for: China’s science, technology, engineering, and mathematics (STEM) research environment: A snapshot
Source: PLoS One. 2018 Apr 3;13(4):e0195347. doi: 10.1371/journal.pone.0195347 (PMC5882148; doi:10.1371/journal.pone.0195347)
Supplement: S1 Appendix — (DOCX) [file pone.0195347.s001.docx]

**Supporting information**

**S1 Appendix. Chinese university rankings by WuShulian and China Alumni Network**

Top 25 Chinese institutions of higher education by 2013 WuShulian rankings; university province, city, and website; corresponding 2013 and 2016 rankings of respective universities by the China Alumni Network. * indicates ranking outside of the top 25 institutions.

| **University name** | **Province** | **City** | **University website** | **2013 WuShulian ranking** | **2013**  **China Alumni Network ranking** | **2016 China Alumni Network ranking** |
| --- | --- | --- | --- | --- | --- | --- |
| Zhejiang University | Zhejiang | Hangzhou | http://www.zju.edu.cn/ | 1 | 4 | 5 |
| Beijing University | Beijing | Beijing | http://www.pku.edu.cn/ | 2 | 1 | 1 |
| Tsinghua University | Beijing | Beijing | http://www.tsinghua.edu.cn/ | 3 | 2 | 2 |
| Shanghai Jiaotong University | Shanghai | Shanghai | http://en.sjtu.edu.cn/ | 4 | 5 | 7 |
| Fudan University | Shanghai | Shanghai | fudan.edu.cn | 5 | 3 | 3 |
| Nanjing University | Jiangsu | Nanjing | www.nju.edu.cn | 6 | 6 | 8 |
| Wuhan University | Hubei | Wuhan | http://www.whu.edu.cn/ | 7 | 9 | 4 |
| Sichuan University | Sichuan | Chengdu | www.scu.edu.cn | 8 | 13 | 14 |
| Sun Yat-sen | Guangdong | Guangzhou | http://www.sysu.edu.cn/ | 9 | 7 | 10 |
| Huazhong University of Science and Technology | Hubei | Wuhan | www.hust.edu.cn | 10 | 11 | 13 |
| Harbin Institute of Technology | Heilongjiang | Harbin | www.hit.edu.cn | 11 | 17 | 21 |
| Shandong University | Shandong | Jinan | http://www.sdu.edu.cn/ | 12 | 15 | 21 |
| Jilin University | Jilin | Changchun | www.jlu.edu.cn | 13 | 8 | 11 |
| Nankai University | Hebei | Tianjin | http://www.nankai.edu.cn/ | 14 | 14 | 16 |
| Xi'an Jiaotong University | Shaanxi | Xian | www.xjtu.edu.cn/en | 15 | 18 | 17 |
| University of Science and Technology of China | Anhui | Hefei | http://en.ustc.edu.cn/ | 16 | 10 | 12 |
| Central South University | Hunan | Changsha | www.csu.edu.cn | 17 | 19 | 18 |
| Southeast University | Jiangsu | Nanjing | seu.edu.cn | 18 | 21 | 24 |
| Renmin University of China | Beijing | Beijing | www.ruc.edu.cn/en | 19 | 12 | 6 |
| Tianjin University | Tianjin | Tianjin | www.tju.edu.cn | 20 | 23 | 20 |
| Dalian University of Technology | Liaoning | Dalian | www.dlut.edu.cn | 21 | 25 | 29* |
| Beijing Normal University | Beijing | Beijing | www.bnu.edu.cn | 22 | 16 | 15 |
| South China University of Technology | Guangdong | Guangzhou | en.scut.edu.cn | 23 | 27* | 31* |
| Tongji University | Shanghai | Shanghai | www.tongji.edu.cn | 24 | 22 | 19 |
| Xiamen University | Fujian | Xiamen | www.xmu.edu.cn | 25 | 20 | 23 |
