# Supplementary material for: China’s science, technology, engineering, and mathematics (STEM) research environment: A snapshot
Source: PLoS One. 2018 Apr 3;13(4):e0195347. doi: 10.1371/journal.pone.0195347 (PMC5882148; doi:10.1371/journal.pone.0195347)
Supplement: S3 Appendix — (DOCX) [file pone.0195347.s003.docx]

**Supporting information**

**S3 Appendix. Survey in Mandarin Chinese**

Full survey in Mandarin Chinese. Coding scheme for each question is provided in parentheses.

Chinese Faculty Survey

Introduction/Consent Page

我们是来自美国加州大学圣塔巴巴拉分校和亚利桑那州立大学,以及英国诺丁汉大学的研究人员。我们主要研究中国,拉丁美洲及欧洲的科技和创新架构。本研究由美国国家科学基金会资助的加州大学圣塔巴巴拉分校纳米科技与社会研究中心(CNS-UCSB: www.cns.ucsb.edu)主持进行. 我们现阶段的研究目标是对中国高等院校总体科研环境及文化进行网上问卷调查。我们的调查对象是在中国排名前25位的高等院校理工院系中从事教学科研工作的教授及副教授群体。   您的参与将为我们提供宝贵的信息来源。因此，我们将不胜感激。   我们真诚的邀请您参与我们关于中国高等教育科研文化的网上问卷调查。完成整个问卷大约需时15-20分钟。问卷内容包括您的科研活动，资金来源，以及您对中国科研环境的看法。 虽然调查人员将有机会获得个人层面的数据，报告将仅在总量上共享。您的回答将受到严格保密，不会以任何方式与您的身份相联。与您的参与没有预见的风险。您的参与是完全自愿的。结果可能会以未来的演讲和出版物的形式呈现给广大公众，但是数据将只在总量报道。   没有正确或错误的答案。你可以自由地跳过你不想回答任何问题，你可以终止调查，你可以在任何时间停止参与这项研究。你可以改变主意，在研究开始后退出这项研究。 如果你对这个研究项目有任何疑问，或如果你认为由于您的参与你可能受到伤害，请联系CNS-UCSB博士后韩雪莹: [email address]. 如果您对自己的权利和参与作为研究对象有任何疑问，请与人类受试者委员会 [phone number] 或 [email address]。或写信给美国加州大学人类受试者委员会，研究办公室，圣巴巴拉，CA 93106-2050   感谢您的参与。

Q1 您所获得的最高学位

- 本科 (1)
- 硕士 (2)
- 博士 (3)
- 其他(请具体说明) (4) ____________________

Q2 您拥有海外学位吗?

- 有 (1)
- 没有 (2)

Answer If 您拥有海外学位吗? 有 Is Selected And 您所获得的最高学位 博士 Is Selected

Q3 请您列出海外博士学位授予的国家

- 美国 (219)
- 加拿大 (220)
- 英国 (221)
- 法国 (222)
- 德国 (223)
- 意大利 (224)
- 澳大利亚 (225)
- 新加坡 (226)
- 日本 (227)
- 菲律宾 (228)
- 韩国 (229)
- 其它欧洲国家 (请具体说明) (230) ____________________
- 其它亚洲国家(请具体说明) (231) ____________________
- 其他 (请具体说明) (232) ____________________

Answer If 您拥有海外学位吗? 有 Is Selected And 您所获得的最高学位 博士 Is Selected

Q4 获取博士学位后，曾经在国外工作过吗?

- 有 (1)
- 沒有 (2)

Answer If 您拥有海外学位吗? 有 Is Selected And 您所获得的最高学位 博士 Is Selected

Q5 您目前在国外教育机构拥有任教职位吗?

- 有 (1)
- 沒有 (2)

Answer If 您拥有海外学位吗? 有 Is Selected And 您所获得的最高学位 博士 Is Not Selected

OR

Answer if您拥有海外学位吗? 有 Is Selected And 您所获得的最高学位 博士 Is Selected And

您目前在国外教育机构拥有任教职位吗? 沒有Is Selected

Q6 您回国前在海外工作了多久?

- < 5 年 (1)
- 5 至 7 年 (2)
- 8 至 10 年 (3)
- 11 至 13 年 (4)
- 14 至 16年 (5)
- 17 至 19 年 (6)
- > 19 年 (7)

Answer If 您拥有海外学位吗? 有 Is Selected

Q7 请问您觉得海外学位给您带来了优势吗?

- 同意 (1)
- 不同意 (2)

Answer If 请问您觉得海外学位给您带来优势吗? 同意 Is Selected

Q8 请问您觉得您的海外学位给您带来了哪些超越国内同等学位的优势? 多项选择

- 声誉 (1)
- 回国后更高的认可度和待遇 (2)
- 更好的相关专业领域关系网 (3)
- 更好的导师和指导 (4)
- 获得更好专业领域教育 (5)
- 更好的就业机会 (6)
- 更好的工资 (7)
- 其他(请具体说明) (8) ____________________

Answer If 请您列出海外博士学位授予的国家 美国 Is Selected

Q9 请问您为何选择在美国求学? 多项选择

- 更好的教育质量 (1)
- 更好的专业研究质量 (2)
- 我可以开展更多的创新性研究 (3)
- 为了获得和具体某个学者的合作机会 (4)
- 为了日后的就业机会 (5)
- 为了能在美国生活 (6)
- 为了能和朋友/家人离得更近一些 (7)
- 为了能有机会体验海外生活 (8)
- 其他(请具体说明) (9) ____________________

Answer If 请您列出海外博士学位授予的国家 美国 Is Selected

Q10 您提到有在美国求学的经历,请问您当初为何决定回到中国. 多项选择

- 我未能获得留在美国所需的机构资助 (1)
- 我的 H-1B 签证申请未获通过 (2)
- 我持有 J1 签证,按照规定毕业后需回国服务 (3)
- 家庭因素 (4)
- 其他个人原因 (5)
- 中国对我个人来说有更多的就业机会 (6)
- 中国对我家人来说有更多的就业机会 (7)
- 我不太适应美国文化 (8)
- 我在中国有更好的专业领域关系网 (9)
- 我不想在美国高等教育机构工作 (10)
- 我希望我的子女能够接受中国教育 (11)
- 增加的时间为在研究 (12)
- 更少的行政责任 (13)
- 其他(请具体说明) (14) ____________________

Answer If 您拥有海外学位吗? 没有 Is Selected And 您所获得的最高学位 博士 Is Selected

Q11 您觉得国外的博士学位将会给你提供任何的优势吗?

- 是 (1)
- 否 (2)

Answer If 您觉得国外的博士学位将会给你提供任何的优势吗? 是 Is Selected

Q12 您觉得外国学历能提供哪种优势? 多项选择

- 声望 (1)
- 一旦回国更能得到认可 (2)
- 更好的专业关系 (3)
- 更好的导师制 (4)
- 更好的教育/知识领域 (5)
- 更好的就业机会 (6)
- 更好的工资 (7)
- 其它(请具体说明) (8) ____________________

Answer If 请您列出海外博士学位授予的国家 美国 Is Not Selected And 您拥有海外学位吗? 有 Is Selected

Q13 您为什么选择到国外学习? 请选择所有适用于您的选答.

- 高质量的教育 (1)
- 高质量的科研领域 (2)
- 可以做更多的创新研究 (3)
- 有机会与特殊人材工作 (4)
- 未来事业机会 (5)
- 想有海外生活经历 (6)
- 有机会接近朋友和家人 (7)
- 其它 (请具体说明) (8) ____________________

Answer If 您拥有海外学位吗? 有 Is Selected And 请您列出海外博士学位授予的国家 美国 Is Not Selected

Q14 您前面说您在外国学习过,请说明您决定回到中国的理由。请选择所有适用于你的选项.

- 得不到留下来的签证 (1)
- 被要求毕业后回国 (2)
- 家庭因素 (3)
- 其他个人原因 (4)
- 中国对我个人来说有更多的就业机会 (5)
- 中国对我家人来说有更多的就业机会 (6)
- 不能适应处国文化 (7)
- 在中国有更好的专业关系 (8)
- 不想在国外高等院校工作 (9)
- 想要孩子受中国教育 (10)
- 在中国院校有更多的科研时间 (11)
- 较少的行政责任 (12)
- 其它 (请具体说明) (13) ____________________

Answer If 您拥有海外学位吗? 有 Is Selected And 请您列出海外博士学位授予的国家 美国 Is Selected

Q15 请您描述你所体验到的中美科学研究环境的最大区别.

Answer If 您拥有海外学位吗? 有 Is Selected And 请您列出海外博士学位授予的国家 美国 Is Not Selected

Q16 请描述一下您所观察到您得到国外学￼位的国家和中国科研或教育环境间的最大的文化差别.

Q17 请问您目前如何选择研究项目? 多项选择

- 自由选择 (1)
- 由系主任提供研究课题 (2)
- 通过大学研究基金项目列表选择 (3)
- 通过省市研究基金项目列表选择 (4)
- 通过国家研究基金项目列表选择 (5)
- 其他(请具体说明) (6) ____________________

Q18 请问您当前的研究项目经费来源是什么? 多项选择

- 中国国家科学基金委员会 (1)
- 科技部 (2)
- 省或地方政府 (3)
- 大学 (4)
- 企业 (5)
- 其他(请具体说明) (6) ____________________

Q19 请问您的研究经费里单纯研究支出所占的百分比是多少?

- 0-25% (1)
- 26-50% (2)
- 51-75% (3)
- 76-100% (4)

Q20请问您目前所有研究项目所获经费资助总额是多少(人民币元)?

- 0-250,000 RMB (1)
- 250,001-500,000 RMB (2)
- 500,001-750,000 RMB (3)
- 750,001-1,000,000 RMB (4)
- 1,000,001 RMB + (5)

Q21请问您有国际研究合作方吗?

- 有 (1)
- 没有 (2)

Answer If 请问您有国际研究合作方吗? 有 Is Selected

Q22 您提到您有国际研究合作方.请问他们来自哪个国家? 请列出:

Answer If 请问您有国际研究合作方吗? 有 Is Selected

Q23 请问您是如何认识这些国外合作者的? 多项选择.

- 通过参加国外举办的专业研讨会 (1)
- 通过参加国内举办的专业研讨会 (2)
- 在海外求学期间认识 (3)
- 通过同事介绍认识 (5)
- 在海外访学期间认识 (4)
- 其他(请具体说明) (6) ____________________

Answer If 请问您有国际研究合作方吗? 有 Is Selected

Q24 您的最长的国际合作有多久?

- < 3 年 (1)
- 3 至 5 年 (2)
- 5 至 7 年 (3)
- 7 至 9 年 (4)
- > 9 年 (5)

Answer If 请问您有国际研究合作方吗? 有 Is Selected

Q25 关于您的最长的国际合作,谁启动的这个合作?

- 您启动的此项合作 (1)
- 您的合作伙伴启动的此项合作 (2)
- 您和您的合作伙伴共同启动的这项合作 (3)
- 第三方(如专业团体,教育机构,投资代理)启动的此次合作 (4)
- 其它(请具体说明) (5) ____________________

Answer If 请问您有国际研究合作方吗? 有 Is Selected

Q26 就那些与国外机构合作研究项目而言,请问经费来源的比例情况是怎样的?

- 全部来自自己 (5)
- 大部分来自于自己一方 (1)
- 大致上自己负责一半,国外合作者负责一半 (2)
- 大部分来自于国外合作者 (3)
- 全部来自合作者 (4)

Q27 请问您有来自于中国国内的合作者吗?

- 有 (1)
- 没有 (2)

Answer If 请问您有来自于中国国内的合作者吗? 有 Is Selected

Q28 您提到有来自中国国内的合作者,请问您是如何认识他们的? 多项选择..

- 通过参加国外举办的专业研讨会 (1)
- 通过参加国内举办的专业研讨会 (2)
- 在中国国内求学期间认识 (3)
- 在访问学者间认识 (4)
- 通过同事介绍认识 (5)
- 院系同事之间 (7)
- 其他(请具体说明) (9) ____________________

Answer If 请问您有国际研究合作方吗? 有 Is Selected And 请问您有来自于中国国内的合作者吗? 有 Is Selected

Q29 您提到有国外及国内的研究合作者, 请问您如何评价两者的合作研究质量

- 和国外学者合作研究质量更高 (1)
- 和国内学者合作研究质量更高 (2)
- 两者没有大的区别 (3)

Q30 请问您平均每年发表多少文章?

- 0 (1)
- 1-3 (2)
- 4-6 (3)
- 7-9 (4)
- 10+ (5)

Q31请问您所在院系或学校有为在国外英文期刊上发表论著提供奖励吗?

- 有 (1)
- 没有 (2)

Answer If 请问您所在院系或学校有为在国外英文期刊上发表论著提供奖励吗? 有 Is Selected

Q32 请您描述在国外英文期刊发表论著的奖励机制:

Q33 请问您有申请过专利吗?

- 有 (1)
- 没有 (2)

Answer If 请问您有申请过专利吗? 有 Is Selected

Q34 有多少专利

- 您申请过? (输入号码) (1) ____________________
- 您被授予?(输入号码) (2) ____________________
- 您目前持有?(输入号码) (3) ____________________

Answer If 请问您有申请过专利吗? 有 Is Selected

Q35 在哪些国家您拥有专利?请选择所有适用于您的选项.

- 中国人民共和国国家知识产权局(SIPO) (1)
- 美国专利和商标局(USPTO) (2)
- 欧洲专利局(EPO) (3)
- 日本专利局 (JPO) (4)
- 其他(请具体说明)) (5) ____________________

Answer If 请问您有申请过专利吗? 有 Is Selected

Q36 请问您所在部门/大学/省/国家部门对拥有专利提供奖励吗?

- 有 (1)
- 没有 (2)

Answer If 请问您有申请过专利吗? 有 Is Selected

Q37 请您表述申请/拥有专利的奖励机制:

Q38 请问您是有否创建过和您的研究领域相关的公司企业吗?

- 有 (1)
- 没有 (2)

Answer If 请问您是有否创建过和您的研究领域相关的公司企业吗? 有 Is Selected

Q39 请问目前企业状况如何?

- 该企业已经停止运营 (1)
- 该企业已经被另外一家私企公司收购了 (2)
- 该企业已经被一家国有企业收购了 (3)
- 该企业还在运营 (4)
- 其他(请具体说明): (5) ____________________

Answer If 请问您是有否创建过和您的研究领域相关的公司企业吗? 有 Is Selected

Q40 哪些资金来源能够使您们的研究商业化? 请选择所有适用于您的选项.

- 个人资金 (1)
- 家庭或朋友 (2)
- 省级政府资金 (3)
- 国家资金 (4)
- 创业投资 (5)
- 其他私人投资(请说明) (6) ____________________
- 其它(请说明): (7) ____________________

Q41 请问您所在院系或学校鼓励教师基于其科研成果创建企业吗?

- 鼓励 (1)
- 不鼓励 (2)
- 中立(我所在院系或学校既不鼓励也不反对教师创立企业) (3)

Answer If 请问您所在院系或学校鼓励教师基于其科研成果创建企业吗? 鼓励 Is Selected

Q42 请描述您所在院系或学校如何鼓励教师创建企业:

Answer If 请问您所在院系或学校鼓励教师基于其科研成果创建企业吗? 不鼓励 Is Selected

Q43 请描述您所在院系或学校如何反对教师创建企业:

Q44 请您选择(按 1-5 的尺度)您对目前职位的满意度

|  | 非常不满意 (1) | (2) | (3) | (4) | 非常满意 (5) |
| --- | --- | --- | --- | --- | --- |
| (Q44) |  |  |  |  |  |

Q45 请您选择(按 1-5 的尺度)您对所任职院系科研环境的满意度

|  | 非常不满意 (1) | (2) | (3) | (4) | 非常满意 (5) |
| --- | --- | --- | --- | --- | --- |
| (Q45) |  |  |  |  |  |

Q46 请您选择(按 1-5 的尺度)您对所在领域总体科研环境的满意度

|  | 非常不满意 (1) | (2) | (3) | (4) | 非常满意 (5) |
| --- | --- | --- | --- | --- | --- |
| (Q46) |  |  |  |  |  |

Q47 请您选择(按 1-5 的尺度)您对中国总体科研环境的满意度

|  | 非常不满意 (1) | (2) | (3) | (4) | 非常满意 (5) |
| --- | --- | --- | --- | --- | --- |
| (Q47) |  |  |  |  |  |

Q48 请您选择(按 1-5 的尺度)您对中国政府目前在促进科研活动方面所起作用的评价

|  | 政府应该比目前降低参与度 (1) | (2) | (3) | (4) | 政府应该比目前提高参与度 (5) |
| --- | --- | --- | --- | --- | --- |
| (Q48) |  |  |  |  |  |

Q49 请您选择(按 1-5 的尺度)您目前的职位对您科研创新性的约束程度

|  | 有很大约束 (1) | (2) | (3) | (4) | 没有任何约束 (5) |
| --- | --- | --- | --- | --- | --- |
| (Q49) |  |  |  |  |  |

Q50 请问您平均每周工作多少小时?

- < 40 小时(1)
- 40-50 小时 (2)
- 50-60 小时 (3)
- >60小时 (4)

Q51 您认为中国目前的科研环境/文化存在任何问题吗?如果有,是什么?

Q52 怎么样的改变(如果应该有所改变的话)能改善中国目前的科研环境/文化?

Q53 您的性别:

- 女 (1)
- 男 (2)

Q54 您的年龄:

- <35 (1)
- 35-45 (2)
- 46-55 (3)
- 56-65 (4)
- >65 (5)

Q55 自获得您的最高学位后,您已经在本行业工作的时长为?

- < 5年 (1)
- 5-10年 (2)
- 11-15 年 (3)
- 16-20 年 (4)
- 21-25 年 (5)
- 26-30 年 (6)
- > 31年 (7)

Q56 您是硕导或博导吗?

- 博导 (1)
- 硕导 (2)
- 同时担任硕导和博导 (3)
- 两者都不是 (4)

Answer If 您是硕导或博导吗? 博导 Is Selected Or

您是硕导或博导吗? 硕导 Is Selected Or

您是硕导或博导吗? 同时担任硕导和博导 Is Selected

Q57 您的研究生通常如何选定其研究课题?

- 我的学生总是或几乎总是自己选定研究课题 (1)
- 我的学生有时候自选课题,有时候我给他们指定课题 (2)
- 我总是或几乎总是给他们指定研究课题 (3)
- 其他情况(请具体说明) (4) ____________________

Q58 2015年, 我们可能访问中国,从事领域调研,我们希望有机会作一次短暂的面谈。请告知您是否愿意参加

- 是 (1)
- 否 (2)

Answer If 2015年;我们可能访问中国,从事领域调研,我们希望有机会作一次短暂的面谈。请告知您是否愿意参加 是 Is Selected

Q59 联系方式

- 姓名: (1) ____________________
- 电子邮件: (2) ____________________
