# Supplementary material for: China’s science, technology, engineering, and mathematics (STEM) research environment: A snapshot
Source: PLoS One. 2018 Apr 3;13(4):e0195347. doi: 10.1371/journal.pone.0195347 (PMC5882148; doi:10.1371/journal.pone.0195347)
Supplement: S4 Appendix — (DOC) [file pone.0195347.s004.doc]

**Supporting information**

**S4 Appendix. Survey in English**

Full survey in English. Coding scheme for each question is provided in parentheses.

We are researchers from the University of California at Santa Barbara, Arizona State University, and University of Nottingham studying science, technology, and innovation frameworks across China, Latin America, and Europe. We are currently conducting an online survey to gain a comprehensive understanding of the research environment and culture at Chinese institutions of higher education. We are contacting all Science, Technology, Engineering, and Mathematics (STEM) professors and associate professors from the top 25 ranked institutions in China to participate in our study. Your responses will remain confidential, and will not be associated in any way with your identity. We plan to publish the findings from this study in a high-impact journal such as Science or Nature to provide a comprehensive assessment of the research community in China. Your participation in this study would be greatly appreciated and provide invaluable information to our research.

We kindly ask for your participation in our online survey regarding the research culture at Chinese institutions of higher education. The survey will last approximately 15-20 minutes and includes questions about your research, funding, and your perceptions of the research environment in China.

Q1 What is the highest degree that you have obtained?

- Bachelor’s (1)
- Master’s (2)
- PhD (3)
- Other (please specify): (4)

Q2 Did you receive your degree from a foreign institution?

- Yes (1)
- No (2)

**Answer If** Did you receive your degree from a foreign institution? Yes **Is Selected And** What is the highest degree that you have obtained? PhD **Is Selected**.

Q3 Please select the country where you received your PhD.

- United States of America (219)
- Canada (220)
- United Kingdom (221)
- France (222)
- Germany (223)
- Italy (224)
- Australia (225)
- Singapore (226)
- Japan (227)
- Philippines (228)
- South Korean (229)
- Other European country (please specify): (230)
- Other Asian country (please specify): (231)
- Other (please specify): (232)

**Answer If** Did you receive your degree from a foreign institution? Yes **Is Selected And** What is the highest degree that you have obtained? PhD **Is Selected**.

Q4 After receiving your PhD, did you remain in the country to work?

- Yes (1)
- No (2)

**Answer If** Did you receive your degree from a foreign institution? Yes **Is Selected And** What is the highest degree that you have obtained? PhD **Is Selected**.

Q5 Do you currently hold a faculty position in China and a foreign institution?

- Yes (1)
- No (2)

**Answer If** Did you receive your degree from a foreign institution? Yes **Is Selected And** What is the highest degree that you have obtained? PhD **Is Not Selected**.

OR

**Answer If** Did you receive your degree from a foreign institution? Yes **Is Selected And** What is the highest degree that you have obtained? PhD **Is Selected** **And** Do you currently hold a faculty position in China and a foreign institution? No Is Selected

Q6 How long did you stay overseas before returning to China?

- <5 years (1)
- 5-7 years (2)
- 8-10 years (3)
- 11-13 years (4)
- 14-16 years (5)
- 17-19 years (6)
- >19 years (7)

**Answer If** Did you receive your degree from a foreign institution? Yes **Is Selected**

Q7 Do you feel that your foreign degree provided you with any advantages?

- Yes (1)
- No (2)

**Answer If** Do you feel that your foreign degree provided you with any advantages? Yes **Is Selected**

Q8 What kind of advantages do you feel your foreign degree provided, beyond what you could have obtained in China? Please select all that apply:

- Prestige (1)
- Better recognition from colleagues in China once you returned (2)
- Better professional network (3)
- Better advisors/mentorship (4)
- Better education/knowledge of your field (5)
- Better job opportunities (6)
- Better pay (7)
- Other (please specify): (8)

**Answer If** Please select the country where you received your PhD. United States of America **Is Selected**

Q9 Why did you choose to study in the US? Please select all that apply:

- Higher quality of education (1)
- Higher quality of research for your field (2)
- I was able to do more innovative research (3)
- Opportunity to work with specific faculty (4)
- Future career opportunities (5)
- Wanted to live in the United States (6)
- Proximity to friends/family (7)
- Wanted to experience living abroad (8)
- Other (please specify): (9)

**Answer If** Please select the country where you received your PhD. United States of America **Is Selected**

Q10 Please indicate your reasons for deciding to return to China. Select all that apply:

- I was not able to get sponsored to stay in the US (1)
- I was not approved for the H-1B visa (2)
- I was on a J1 visa and was required to return to China upon graduation (3)
- Family (4)
- Other personal reasons (5)
- More job opportunities for myself in China (6)
- More job opportunities for my family in China (7)
- I did not adjust well to American culture (8)
- I had a better professional network in China (9)
- I did not want to work in an American higher education institution (10)
- I wanted my children to receive a Chinese education (11)
- Increased time for research (12)
- Fewer administrative responsibilities at Chinese institutions (13)
- Other (please specify): (14)

**Answer If** Did you receive your degree from a foreign institution? No **Is Selected And** What is the highest degree that you have obtained? PhD **Is Selected**.

Q11 Do you feel that a foreign PhD degree would have provide you with any advantages?

- Yes (1)
- No (2)

**Answer If** Do you feel that a foreign PhD degree would have provide you with any advantages? Yes **Is Selected**

Q12 What kind of advantages do you a foreign degree would have provided? Please select all that apply:

- Prestige (1)
- Better recognition from colleagues in China once you returned (2)
- Better professional network (3)
- Better advisors/mentorship (4)
- Better education/knowledge of your field (5)
- Better job opportunities (6)
- Better pay (7)
- Other (please specify): (8)

**Answer If** Please select the country where you received your PhD. United States of America **Is Not Selected And** Did you receive your degree from a foreign institution? Yes **Is Selected**

Q13 Why did you choose to study abroad? Please select all that apply:

- Higher quality of education (1)
- Higher quality of research for your field (2)
- I was able to do more innovative research (3)
- Opportunity to work with specific faculty (4)
- Future career opportunities (5)
- Wanted to experience living abroad (6)
- Proximity to friends/family (7)
- Other (please specify): (8)

**Answer If** Did you receive your degree from a foreign institution? Yes **Is Selected** **AND** Please select the country where you received your PhD. United States of America **Is Not Selected**

Q14 You previously indicated that you have studied in a foreign country, please indicate your reasons for deciding to return to China. Please select all that apply:

- I was not able to obtain a visa to stay (1)
- I was required to return to China upon graduation (2)
- Family (3)
- Other personal reasons (4)
- More job opportunities for myself in China (5)
- More job opportunities for my family in China (6)
- I did not adjust well to the foreign culture (7)
- I had a better professional network in China (8)
- I did not want to work in a foreign higher education institution (9)
- I wanted my children to receive a Chinese education (10)
- Increased time for research (11)
- Fewer administrative responsibilities at Chinese institutions (12)
- Other (please specify): (13)

**Answer If** Did you receive your degree from a foreign institution? Yes **Is Selected** **AND** Please select the country where you received your PhD. United States of America **Is Selected**

Q15 Please describe the biggest differences you have observed between American and Chinese research/education environments.

**Answer If** Did you receive your degree from a foreign institution? Yes **Is Selected** **AND** Please select the country where you received your PhD. United States of America **Is Not Selected**

Q16 Please describe the biggest differences you have observed between the academic culture where you received your PhD and the Chinese research/education environment.

Q17 How do you choose your research projects? Please select all that apply:

- Through self selection (1)
- Topic provided by department chair (2)
- Topic selected through a university funding list (3)
- Topic selected through a provincial funding list (4)
- Topic selected through a national funding list (5)
- Other (please specify): (6)

Q18 From what sources are your current research projects funded? Please select all that apply:

- National Natural Science Foundation of China (1)
- Ministry of Science and Technology (2)
- Provincial or local government (3)
- University (4)
- Private company or companies (5)
- Other (please specify): (6)

Q19 What percentage of your funding goes towards research?

- 0-25% (1)
- 26-50% (2)
- 51-75% (3)
- >75% (4)

Q20 How much funding (in RMB) do you currently have for all of your research projects?

- 0-250,000 RMB (1)
- 250,001-500,000 RMB (2)
- 500,001-750,000 RMB (3)
- 750,001-1,000,000 RMB (4)
- 1,000,001 RMB + (5)

Q21 Do you have any international collaborators?

- Yes (1)
- No (2)

**Answer If** Do you have any international collaborators? Yes **Is Selected**

Q22 What countries are your international collaborators from? Please list:

**Answer If** Do you have any international collaborators? Yes **Is Selected**

Q23 How did you meet these international collaborators? Please select all that apply:

- At a professional conference outside China (1)
- At a professional conference in China (2)
- While you were studying abroad (3)
- While you were visiting professor/scholar (4)
- Through a colleague (5)
- Other (please specify): (6)

**Answer If** Do you have any international collaborators? Yes **Is Selected**

Q24 How long was your longest international collaboration?

- <3 years (1)
- 3-5 years (2)
- 5-7 years (3)
- 7-9 years (4)
- >9 years (5)

**Answer If** Do you have any international collaborators? Yes **Is Selected**

Q25 Regarding your longest international collaboration, who initiated the collaboration?

- You initiated the collaboration (1)
- Your collaborator initiated the collaboration (2)
- You and your collaborator equally initiated the collaboration (3)
- A third party (e.g., professional society, institution, funding agency) initiated the collaboration (4)
- Other (please specify): (5)

**Answer If** Do you have any international collaborators? Yes **Is Selected**

Q26 For research projects that are done in conjunction with international collaborators, how much of the funding comes from you and how much of the funding comes from your collaborator?

- I provide almost all of the funding (5)
- I provide more than 50% of the funding (1)
- I provide roughly half of the funding and my international collaborator provides the other half (2)
- My international collaborator provides more than 50% of the funding (3)
- My international collaborator provides almost all of the funding (4)

Q27 Do you have any collaborators within China?

- Yes (1)
- No (2)

**Answer If** Do you have any collaborators within China? Yes **Is Selected**

Q28 You stated in the previous question that you have collaborators within China, how did you meet these collaborators? Please select all that apply.

- At a professional conference outside China (1)
- At a professional conference in China (2)
- During your studies in China (3)
- While you were a visiting professor/scholar (4)
- Through a colleague (5)
- Through a departmental colleague (7)
- Other (please specify): (9)

**Answer If** Do you have any international collaborators? Yes **Is Selected** **And** Do you have any collaborators within China? Yes **Is Selected**

Q29 You have stated that you collaborate with both international and domestic researchers, do you feel that the quality of research you engage in is higher with international or domestic collaborators?

- Higher quality of research with international collaborators (1)
- Higher quality of research with domestic collaborators (2)
- Equal quality of research between international and domestic collaborators (3)

Q30 On average, how many publications do you publish annually?

- 0 (1)
- 1-3 (2)
- 4-6 (3)
- 7-9 (4)
- 10+ (5)

Q31 Does your university/department offer any incentives for publishing in an English based foreign journal?

- Yes (1)
- No (2)

**Answer If** Does your university/department offer any incentives for publishing in an English based foreign journal? Yes **Is Selected**

Q32 Please describe the incentives that are offered for publishing in an English based foreign journal.

Q33 Do you hold any patents?

- Yes (1)
- No (2)

**Answer If** Do you hold any patents? Yes **Is Selected**

Q34 How many patents

- Have you applied for? (1)
- Have you been granted? (2)
- Do you currently hold? (3)

**Answer If** Do you hold any patents? Yes **Is Selected**

Q35 In which countries do you hold patents? Please select all that apply:

- State Intellectual Property Office of the People’s Republic of China (SIPO) (1)
- United States Patent and Trademark Office (USPTO) (2)
- European Patent Office (EPO) (3)
- Japanese Patent Office (JPO) (4)
- Other (please specify): (5)

**Answer If** Do you hold any patents? Yes **Is Selected**

Q36 Does your department/institution/province/federal agency offer any incentives for creating/owning patents?

- Yes (1)
- No (2)

**Answer If** Do you hold any patents? Yes **Is Selected**

Q37 Please describe the incentives that are offered for creating/owning patents:

Q38 Have you ever created a start-up company related to your field of research?

- Yes (1)
- No (2)

**Answer If** Have you ever created a start-up company related to your field of research? Yes **Is Selected**

Q39 What is the current status of this start-up?

- The company is no longer running (1)
- The company was bought by another public company (2)
- The company was bought by a state-owned company (3)
- The company is still in existence (4)
- Other (please specify): (5)

**Answer If** Have you ever created a start-up company related to your field of research? Yes **Is Selected**

Q40 What funding sources enabled you to commercialize your research? Please select all that apply:

- Personal finances (1)
- Family and friends (2)
- Provincial funding (3)
- Federal funding (4)
- Venture capital funding (5)
- Other privatized funding (please specify): (6)
- Other (please specify): (7)

Q41 Does your department/university encourage faculty members to create start-up companies based on their research?

- My department/university **encourages** faculty members to create start-ups (1)
- My department/university **discourages** faculty members to create start-ups (2)
- Neither, my department/university does not encourage or discourage faculty members from creating start-ups (3)

**Answer If** Does your department/university encourage faculty members to create start-up companies based on their research? My department/university encourages faculty members to create start-ups **Is Selected**

Q42 Please indicate in which ways your department/university encourage faculty members to create start-up companies.

**Answer If** Does your department/university encourage faculty members to create start-up companies based on their research? My department/university discourages faculty members to create start-ups **Is Selected**

Q43 Please indicate in which ways your department/university discourage faculty members to create start-up companies.

Q44 On a scale of 1 to 5, how satisfied are you at your current position? (1=very unsatisfied, 5=very satisfied)

Q45 On a scale of 1 to 5, how satisfied are you with the research culture in your department? (1=very unsatisfied, 5=very satisfied)

Q46 On a scale of 1 to 5, how satisfied are you with the overall research culture in field? (1=very unsatisfied, 5=very satisfied)

Q47 On a scale of 1 to 5, how satisfied are you with the overall research culture in China? (1=very unsatisfied, 5=very satisfied)

Q48 On a scale of 1 to 5, how do you feel about the current role of the Chinese central government in supporting research activities? (1=The government should be much less involved than it is currently, 5=The government should be much more involved than it is currently)

Q49 On a scale of 1 to 5, how creatively limited are you by your current position? (1=very limited, 5=not at all limited)

Q50 On average, how many hours do you work per week?

- <40 hours (1)
- 40-50 hours (2)
- 50-60 hours (3)
- >60 hours (4)

Q51 What challenges (if any) do you think exist in China’s current research environment/culture?

Q52 What changes (if any) do you think would improve the research environment/culture in China?

Q53 What is your gender?

- Female (1)
- Male (2)

Q54 Please select the age group to which you belong:

- <35 (1)
- 35-45 (2)
- 45-55 (3)
- 55-65 (4)
- >65 (5)

Q55 How many years after receiving your terminal degree have you worked in your current field?

- < 5 years (1)
- 5-10 years (2)
- 11-15 years (3)
- 16-20 years (4)
- 21-25 years (5)
- 26-30 years (6)
- > 31 years (7)

Q56 Do you advise any masters or doctoral students?

- I advise doctoral students (1)
- I advise masters students (2)
- I advise both masters and doctoral students (3)
- I do not advise any graduate students (4)

**Answer If** Do you advise any masters or doctoral students? I advise doctoral students **Is Selected** **Or** I advise masters students **Is Selected Or** I advise both masters and doctoral students **Is Selected**

Q57 How do the students you advise usually select their thesis/dissertation topic?

- The students always or almost always choose the topics themselves (1)
- The students sometimes choose their topics, and sometimes I give them the topic that they will be working on (2)
- I always or almost always give them the topic that they will be working on (3)
- Other (please specify): (4)

Q58 We may be visiting China in 2015 to conduct field research and we would welcome the opportunity to have a brief follow-up interview. Please let us know if you are willing to do so.

- Yes (1)
- No (2)

**Answer If** We may be visiting China in 2015 to conduct field research and we would welcome the opportunity to have a brief follow-up interview. Please let us know if you are willing to do so. Yes **Is Selected**

Q59 Contact Form

- Name: (1)
- Email: (2)
