# Supplementary material for: China’s science, technology, engineering, and mathematics (STEM) research environment: A snapshot
Source: PLoS One. 2018 Apr 3;13(4):e0195347. doi: 10.1371/journal.pone.0195347 (PMC5882148; doi:10.1371/journal.pone.0195347)
Supplement: S5 Appendix — (DOCX) [file pone.0195347.s005.docx]

**Supporting information**

**S5 Appendix. Calculations and results for survey non-response bias**

For single-answer questions, Pearson’s chi-squared test of independence was used to determine if there were significant differences in responses between early and late respondents. For response where the sample size was low (i.e., ≤5), Fisher’s Exact Test was used. For multiple-answer questions, a general linear model with a binomial distribution was used for each answer choice where *time of response* (early vs. late) was a fixed, independent variable and the dichotomous response (0 if the answer choice was not selected, and 1 if the answer choice was selected) was the dependent variable. We considered the response to be significantly different between early and late respondents when the P-value < 0.05. A contingency table or frequency table is provided for each multiple-choice question. For survey logic, please refer to the full survey in S3 Appendix (Mandarin Chinese) or S4 Appendix (English). * indicates significant differences in response between early and late respondents.

**Question 1. What is the highest degree that you have obtained?**

Contingency table

|  | Early respondents | Late respondents |
| --- | --- | --- |
| Bachelor’s | 5 | 0 |
| Master’s | 13 | 10 |
| PhD | 484 | 216 |
| Other | 1 | 2 |

Fisher’s Exact Test

P=0.12

**Question 2: Did you receive your PhD from a foreign institution?**

Contingency table

|  | Early respondents | Late respondents |
| --- | --- | --- |
| Yes | 77 | 45 |
| No | 426 | 183 |

Pearson’s chi-squared test with Yates’ continuity correction

Χ^2^_1_ = 1.91, P=0.17

**Question 3: Please select the country where you received your PhD.**

**Question 4: After receiving your PhD, did you remain in the country to work?**

Contingency table

|  | Early respondents | Late respondents |
| --- | --- | --- |
| Yes | 64 | 35 |
| No | 13 | 9 |

Pearson’s chi-squared test with Yates’ continuity correction

Χ^2^_1_ = 0.06, P=0.81

**Question 5: Do you currently also hold a faculty position in a foreign institution?**

Contingency table

|  | Early respondents | Late respondents |
| --- | --- | --- |
| Yes | 15 | 8 |
| No | 62 | 36 |

Pearson’s chi-squared test with Yates’ continuity correction

Χ^2^_1_ = 0.0, P=1.0

**Question 6: How long did you stay overseas before returning to China?**

Contingency table

|  | Early respondents | Late respondents |
| --- | --- | --- |
| < 5 years | 38 | 20 |
| 5-7 years | 10 | 8 |
| 8-10 years | 5 | 5 |
| 11-13 years | 2 | 0 |
| 14-16 years | 2 | 0 |
| 17-19 years | 2 | 1 |
| > 19 years | 2 | 1 |

Fisher’s Exact Test

P=0.84

**Question 7: Do you feel that your foreign degree provided you with any advantages?**

Contingency table

|  | Early respondents | Late respondents |
| --- | --- | --- |
| Yes | 66 | 36 |
| No | 11 | 8 |

Pearson’s chi-squared test with Yates’ continuity correction

Χ^2^_1_ = 0.09, P=0.76

**Question 8: What kind of advantages do you feel your foreign degree provided, beyond what you could have obtained in China? Please select all that apply.**

Frequency table

|  | Early respondents (N=66) | Late respondents (N=36) |
| --- | --- | --- |
| Prestige | 35 | 15 |
| Better recognition from colleagues in China | 40 | 19 |
| Better professional networks | 17 | 10 |
| Better advisors/mentorship | 32 | 16 |
| Better education/knowledge of your field | 44 | 28 |
| Better job opportunities | 26 | 14 |
| Better pay | 12 | 6 |
| Other (please specify) | 0 | 1 |

Type-III analysis of deviance results from general linear model

| **Dependent variable** | **Likelihood Ratio Χ^2^_1_** | **P-value** |
| --- | --- | --- |
| Prestige | 1.21 | 0.27 |
| Better recognition from colleagues in China | 0.58 | 0.45 |
| Better professional networks | 0.05 | 0.83 |
| Better advisors/mentorship | 0.15 | 0.70 |
| Better education/knowledge of your field | 1.42 | 0.23 |
| Better job opportunities | 0.002 | 0.96 |
| Better pay | 0.04 | 0.85 |
| Other (please specify) | 2.10 | 0.15 |

**Question 9: Why did you choose to study in the US? Please select all that apply.**

Frequency table

|  | Early respondents | Late respondents |
| --- | --- | --- |
| Higher quality of education | 24 | 13 |
| Higher quality of research for your field | 25 | 13 |
| Wanted to experience living abroad | 13 | 5 |
| Do more innovative research | 2 | 0 |
| Opportunity to work with specific faculty | 8 | 4 |
| Future career opportunities | 7 | 0 |
| Wanted to live in the US | 1 | 0 |
| Proximity to friends/family | 19 | 8 |
| Other | 1 | 0 |

Type-III analysis of deviance results from general linear model

| **Dependent variable** | **Likelihood Ratio Χ^2^_1_** | **P-value** |
| --- | --- | --- |
| Higher quality of education | 1.21 | 0.27 |
| Higher quality of research for your field | 5.95 | 0.01* |
| Do more innovative research | 0.003 | 0.95 |
| Opportunity to work with specific faculty | 1.36 | 0.24 |
| Future career opportunities | 0.20 | 0.65 |
| Wanted to live in the US | 5.13 | 0.02* |
| Proximity to friends/family | 0.67 | 0.41 |
| Wanted to experience living abroad | 0.06 | 0.81 |
| Other | 0.67 | 0.41 |

***Question 11: Do you feel that a foreign PhD degree would have provided you with any advantages?**

Contingency table

|  | Early respondents | Late respondents |
| --- | --- | --- |
| Yes | 336 | 127 |
| No | 60 | 40 |

Pearson’s chi-squared test with Yates’ continuity correction

Χ^2^_1_ = 5.6, P=0.02

**Question 12: What kind of advantages do you a foreign degree would have provided? Please select all that apply.**

Frequency table

|  | Early respondents (N=336) | Late respondents (N=127) |
| --- | --- | --- |
| Prestige | 186 | 59 |
| Better recognition from colleagues in China | 240 | 78 |
| Better professional network | 151 | 51 |
| Better advisors/mentorship | 89 | 28 |
| Better education/knowledge of your field | 215 | 79 |
| Better job opportunities | 185 | 55 |
| Better pay | 137 | 45 |
| Other | 19 | 7 |

Type-III analysis of deviance results from general linear model

| **Dependent variable** | **Likelihood Ratio Χ^2^_1_** | **P-value** |
| --- | --- | --- |
| Higher quality of education | 1.21 | 0.27 |
| Higher quality of research for your field | 5.95 | 0.01* |
| Do more innovative research | 0.003 | 0.95 |
| Opportunity to work with specific faculty | 1.36 | 0.24 |
| Future career opportunities | 0.20 | 0.65 |
| Wanted to live in the US | 5.13 | 0.02* |
| Proximity to friends/family | 0.67 | 0.41 |
| Wanted to experience living abroad | 0.06 | 0.81 |
| Other | 0.67 | 0.41 |

**Question 17: How do you choose your research projects? Please select all that apply.**

Contingency table

|  | Early respondents | Late respondents |
| --- | --- | --- |
| Through self selection | 333 | 160 |
| Provided by department chair | 10 | 3 |
| Through a university funding list | 96 | 35 |
| Through a provincial funding list | 166 | 78 |
| Trough a national funding list | 359 | 157 |
| Other | 51 | 21 |

Fisher’s Exact Test

P=0.83

**Question 18: From what sources are your current research projects funded? Please select all that apply.**

Frequency table

| **Dependent variable** | **Early respondents** | **Late respondents** |
| --- | --- | --- |
| National Natural Science Foundation of China | 407 | 184 |
| Ministry of Science and Technology | 200 | 90 |
| Provincial or local government | 201 | 100 |
| University | 156 | 60 |
| Private company or companies | 212 | 102 |
| Other (please specify) | 24 | 16 |

Type-III analysis of deviance results from general linear model

| **Dependent variable** | **Likelihood Ratio Χ^2^_1_** | **P-value** |
| --- | --- | --- |
| National Natural Science Foundation of China | 0.005 | 0.95 |
| Ministry of Science and Technology | 0.005 | 0.94 |
| Provincial or local government | 0.98 | 0.32 |
| University | 1.7 | 0.19 |
| Private company or companies | 0.43 | 0.51 |
| Other (please specify) | 1.47 | 0.23 |

**Question 19: What percentage of your funding goes towards research?**

Contingency table

|  | Early respondents | Late respondents |
| --- | --- | --- |
| 0-25% | 13 | 2 |
| 26-50% | 72 | 40 |
| 51-75% | 253 | 112 |
| 76-100% | 158 | 73 |

Fisher’s Exact Test

P=0.36

**Question 20: How much funding (in RMB) do you currently have for all of your research projects?**

Contingency table

|  | Early respondents | Late respondents |
| --- | --- | --- |
| 0-250,000 RMB | 52 | 31 |
| 250,001-500,000 RMB | 84 | 31 |
| 500,001-750,000 RMB | 69 | 28 |
| 750,001-1,000,000 RMB | 76 | 37 |
| 1,000,001 RMB + | 211 | 98 |

Pearson’s chi-squared test

Χ^2^_4_ = 2.82, P=0.59

**Question 21: Do you have any international collaborators?**

Contingency table

|  | Early respondents | Late respondents |
| --- | --- | --- |
| Yes | 241 | 118 |
| No | 262 | 110 |

Pearson’s chi-squared test

Χ^2^_1_ = 0.78, P=0.38

**Question 23: How did you meet these international collaborators? Please select all that apply.**

Frequency table

| **Dependent variable** | **Early respondents** | **Late respondents** |
| --- | --- | --- |
| At a professional conference outside China | 94 | 52 |
| At a professional conference in China | 45 | 27 |
| While studying abroad | 56 | 32 |
| As a visiting professor/scholar | 155 | 65 |
| Through a colleague | 88 | 42 |
| Other (please specify) | 37 | 15 |

Type-III analysis of deviance results from general linear model

| **Dependent variable** | **Likelihood Ratio Χ^2^_1_** | **P-value** |
| --- | --- | --- |
| At a professional conference outside China | 1.64 | 0.20 |
| At a professional conference in China | 1.44 | 0.23 |
| While studying abroad | 1.22 | 0.27 |
| As a visiting professor/scholar | 0.40 | 0.53 |
| Through a colleague | 0.09 | 0.76 |
| Other (please specify) | 0.15 | 0.70 |

**Question 24: How long was your longest international collaboration?**

Contingency table

|  | Early respondents | Late respondents |
| --- | --- | --- |
| <3 years | 70 | 43 |
| 3-5 years | 75 | 34 |
| 5-7 years | 26 | 12 |
| 7-9 years | 20 | 6 |
| >9 years | 49 | 22 |

Pearson’s chi-squared test

Χ^2^_4_ = 2.79, P=0.59

**Question 25: Regarding your longest international collaboration, who initiated the collaboration?**

Contingency table

|  | Early respondents | Late respondents |
| --- | --- | --- |
| You initiated the collaboration | 70 | 29 |
| Your collaborator initiated the collaboration | 30 | 23 |
| You and your collaborator equally initiated the collaboration | 122 | 58 |
| A third party (e.g., professional society, institution, funding agency) initiated the collaboration + Other (please specify) | 18 | 6 |

Pearson’s chi-squared test

Χ^2^_3_ = 3.95, P=0.27

**Question 26: For research projects that are done in conjunction with international collaborators, how much of the funding comes from you and how much of the funding comes from your collaborator?**

Contingency table

|  | Early respondents | Late respondents |
| --- | --- | --- |
| I provide almost all of the funding | 35 | 15 |
| I provide more than 50% of the funding | 83 | 34 |
| I provide roughly half of the funding and my international collaborator provides the other half | 74 | 48 |
| My international collaborator provides more than 50% of the funding | 28 | 9 |
| My international collaborator provides almost all of the funding | 13 | 10 |

Pearson’s chi-squared test

Χ^2^_4_ = 5.62, P=0.23

**Question 27: Do you have any collaborators within China?**

Contingency table

|  | Early respondents | Late respondents |
| --- | --- | --- |
| Yes | 430 | 195 |
| No | 73 | 33 |

Pearson’s chi-squared test

Χ^2^_1_ = 0.00, P=1.00

**Question 28: You stated in the previous question that you have collaborators within China, how did you meet these collaborators? Please select all that apply.**

Frequency table

| **Dependent variable** | **Early respondents** | **Late respondents** |
| --- | --- | --- |
| At a professional conference outside China | 58 | 32 |
| At a professional conference in China | 243 | 118 |
| During your studies in China | 190 | 71 |
| As a visiting professor/scholar | 52 | 23 |
| Through a colleague | 234 | 84 |
| Through a departmental colleague | 228 | 97 |
| Other (please specify) | 35 | 15 |

Type-III analysis of deviance results from general linear model

| **Dependent variable** | **Likelihood Ratio Χ^2^_1_** | **P-value** |
| --- | --- | --- |
| At a professional conference outside China | 0.89 | 0.34 |
| At a professional conference in China | 0.74 | 0.39 |
| During your studies in China | 3.04 | 0.08 |
| As a visiting professor/scholar | 0.01 | 0.92 |
| Through a colleague | 6.03 | 0.01* |
| Through a departmental colleague | 0.49 | 0.48 |
| Other (please specify) | 0.04 | 0.85 |

**Question 29: You have stated that you collaborate with both international and domestic researchers, do you feel that the quality of research you engage in is higher with international or domestic collaborators?**

Contingency table

|  | Early respondents | Late respondents |
| --- | --- | --- |
| Higher quality of research with international collaborators | 82 | 38 |
| Higher quality of research with domestic collaborators | 13 | 7 |
| Equal quality of research between international and domestic collaborators | 122 | 64 |

Pearson’s chi-squared test

Χ^2^_2_ = 0.27, P=0.87

**Question 30: On average, how many publications do you publish annually?**

Contingency table

|  | Early respondents | Late respondents |
| --- | --- | --- |
| 0 | 6 | 1 |
| 1-3 | 271 | 117 |
| 4-6 | 141 | 71 |
| 7-9 | 52 | 17 |
| 10+ | 30 | 21 |

Fisher’s Exact Test

P=0.27

**Question 31: Does your university/department offer any incentives for publishing in an English based foreign journal?**

Contingency table

|  | Early respondents | Late respondents |
| --- | --- | --- |
| Yes | 386 | 164 |
| No | 117 | 64 |

Pearson’s chi-squared test

Χ^2^_1_ = 1.70, P=0.19

**Question 33: Do you hold any patents?**

Contingency table

|  | Early respondents | Late respondents |
| --- | --- | --- |
| Yes | 305 | 145 |
| No | 198 | 83 |

Pearson’s chi-squared test

Χ^2^_1_ = 0.46, P=0.50

**Question 35: In which countries do you hold patents? Please select all that apply.**

Frequency table

|  | Early respondents | Late respondents |
| --- | --- | --- |
| State Intellectual Property Office of the People’s Republic of China (SIPO) | 288 | 138 |
| United States Patent and Trademark Office (USPTO) | 36 | 20 |
| European Patent Office (EPO) | 11 | 3 |
| Japanese Patent Office (JPO) | 14 | 3 |
| Other (please specify) | 7 | 2 |

Type-III analysis of deviance results from general linear model

|  | Likelihood ratio Χ^2^_1_ | P-value |
| --- | --- | --- |
| State Intellectual Property Office of the People’s Republic of China (SIPO) | 0.11 | 0.74 |
| United States Patent and Trademark Office (USPTO) | 0.35 | 0.55 |
| European Patent Office (EPO) | 0.83 | 0.36 |
| Japanese Patent Office (JPO) | 1.91 | 0.17 |
| Other (please specify) | 0.45 | 0.50 |

**Question 36: Does your department/institution/province/federal agency offer any incentives for creating/owning patents?**

Contingency table

|  | Early respondents | Late respondents |
| --- | --- | --- |
| Yes | 167 | 75 |
| No | 133 | 69 |

Pearson’s chi-squared test

Χ^2^_1_ = 0.37, P=0.54

**Question 38: Have you ever created a start-up company related to your field of research?**

Contingency table

|  | Early respondents | Late respondents |
| --- | --- | --- |
| Yes | 67 | 38 |
| No | 428 | 187 |

Pearson’s chi-squared test

Χ^2^_1_ = 1.14, P=0.29

**Question 39: What is the current status of this start-up?**

Contingency table

|  | Early respondents | Late respondents |
| --- | --- | --- |
| The company is no longer running | 12 | 6 |
| The company was bought by another public company | 8 | 2 |
| The company was bought by a state-owned company | 1 | 0 |
| The company is still in existence | 44 | 29 |
| Other (please specify) | 2 | 1 |

Fisher’s Exact Test

P=0.79

**Question 40: What funding sources enabled you to commercialize your research? Please select all that apply.**

Frequency table

|  | Early respondents | Late respondents |
| --- | --- | --- |
| Personal finances | 39 | 20 |
| Family and friends | 19 | 8 |
| Provincial funding | 20 | 18 |
| Federal funding | 25 | 12 |
| Venture capital funding | 48 | 25 |
| Other privatized funding (please specify) | 14 | 7 |
| Other (please specify) | 3 | 1 |

Type-III analysis of deviance results from general linear model

|  | Likelihood ratio Χ^2^_1_ | P-value |
| --- | --- | --- |
| Personal finances | 0.31 | 0.58 |
| Family and friends | 0.69 | 0.41 |
| Provincial funding | 3.19 | 0.07 |
| Federal funding | 0.35 | 0.55 |
| Venture capital funding | 0.39 | 0.53 |
| Other privatized funding (please specify) | 0.09 | 0.76 |
| Other (please specify) | 0.24 | 0.63 |

**Question 41: Does your department/university encourage faculty members to create start-up companies based on their research?**

Contingency table

|  | Early respondents | Late respondents |
| --- | --- | --- |
| My department/university **encourages** faculty members to create start-ups | 79 | 42 |
| My department/university **discourages** faculty members to create start-ups | 87 | 30 |
| Neither, my department/university does not encourage or discourage faculty members from creating start-ups | 329 | 152 |

Pearson’s chi-squared test

Χ^2^_2_ = 2.42, P=0.30

**Question 44: On a scale of 1 to 5, how satisfied are you at your current position?**

Contingency table

|  | Early respondents | Late respondents |
| --- | --- | --- |
| 1 = very unsatisfied | 21 | 10 |
| 2 = unsatisfied | 44 | 18 |
| 3 = neither unsatisfied nor satisfied | 159 | 74 |
| 4 = satisfied | 215 | 89 |
| 5 = very satisfied | 59 | 34 |

Pearson’s chi-squared test

Χ^2^_4_ = 1.95, P=0.74

**Question 45: On a scale of 1 to 5, how satisfied are you with the research culture in your department?**

Contingency table

|  | Early respondents | Late respondents |
| --- | --- | --- |
| 1 = very unsatisfied | 50 | 19 |
| 2 = unsatisfied | 102 | 44 |
| 3 = neither unsatisfied nor satisfied | 187 | 74 |
| 4 = satisfied | 136 | 73 |
| 5 = very satisfied | 21 | 12 |

Pearson’s chi-squared test

Χ^2^_4_ = 3.25, P=0.52

**Question 46: On a scale of 1 to 5, how satisfied are you with the overall research culture in field?**

Contingency table

|  | Early respondents | Late respondents |
| --- | --- | --- |
| 1 = very unsatisfied | 34 | 16 |
| 2 = unsatisfied | 106 | 38 |
| 3 = neither unsatisfied nor satisfied | 196 | 87 |
| 4 = satisfied | 15 | 8 |
| 5 = very satisfied | 142 | 75 |

Pearson’s chi-squared test

Χ^2^_4_ = 2.87, P=0.58

**Question 47: On a scale of 1 to 5, how satisfied are you with the overall research culture in China?**

Contingency table

|  | Early respondents | Late respondents |
| --- | --- | --- |
| 1 = very unsatisfied | 64 | 27 |
| 2 = unsatisfied | 154 | 56 |
| 3 = neither unsatisfied nor satisfied | 189 | 92 |
| 4 = satisfied | 84 | 46 |
| 5 = very satisfied | 7 | 2 |

Fisher’s Exact Test

P=0.44

**Question 48: On a scale of 1 to 5, how do you feel about the current role of the Chinese central government in supporting research activities?**

Contingency table

|  | Early respondents | Late respondents |
| --- | --- | --- |
| 1 = The government should be much less involved than it is currently | 101 | 35 |
| 2 = The government should be less involved than it is currently | 111 | 43 |
| 3 = The government should maintain its current level of involvement | 159 | 88 |
| 4 = The government should be more involved than it is currently | 90 | 50 |
| 5 = The government should be much more involved than it is currently | 35 | 9 |

Pearson’s chi-squared test

Χ^2^_4_ = 8.61, P=0.07

**Question 49: On a scale of 1 to 5, how creatively limited are you by your current position?**

Contingency table

|  | Early respondents | Late respondents |
| --- | --- | --- |
| 1 = very limited | 62 | 22 |
| 2 = limited | 86 | 27 |
| 3 = neither limited nor not limited | 133 | 71 |
| 4 = not limited | 120 | 58 |
| 5 = very not limited | 97 | 47 |

Pearson’s chi-squared test

Χ^2^_4_ = 5.33, P=0.26

**Question 50: On average, how many hours do you work per week?**

Contingency table

|  | Early respondents | Late respondents |
| --- | --- | --- |
| <40 hours | 33 | 10 |
| 40-50 hours | 144 | 72 |
| 50-60 hours | 166 | 72 |
| >60 hours | 154 | 71 |

Pearson’s chi-squared test

Χ^2^_3_ = 1.84, P=0.61

**Question 53: What is your gender?**

Contingency table

|  | Early respondents | Late respondents |
| --- | --- | --- |
| Female | 89 | 47 |
| Male | 409 | 177 |

Pearson’s chi-squared test

Χ^2^_1_ = 0.79, P=0.38

**Question 54: Please select the age group to which you belong.**

Contingency table

|  | Early respondents | Late respondents |
| --- | --- | --- |
| < 35 | 53 | 16 |
| 35-45 | 248 | 95 |
| 45-55 | 154 | 90 |
| 55-65 | 36 | 21 |
| > 65 | 8 | 3 |

Fisher’s Exact Test

P=0.07

***Question 55: How many years after receiving your terminal degree have you worked in your current field?**

Contingency table

|  | Early respondents | Late respondents |
| --- | --- | --- |
| < 5 years | 33 | 18 |
| 5-10 years | 171 | 62 |
| 11-15 years | 161 | 55 |
| 16-20 years | 66 | 44 |
| 21-25 years | 34 | 24 |
| 26-30 years | 21 | 15 |
| > 31 years | 13 | 6 |

Pearson’s chi-squared test

Χ^2^_6_ = 14.65, P=0.02

**Question 56: Do you advise any masters or doctoral students?**

Contingency table

|  | Early respondents | Late respondents |
| --- | --- | --- |
| I advise doctoral students | 64 | 45 |
| I advise masters students | 215 | 83 |
| I advise both masters and doctoral students | 209 | 95 |
| I do not advise any graduate students | 10 | 2 |

Fisher’s Exact Test

P=0.05

**Question 57: How do the students you advise usually select their thesis/dissertation topic?**

Contingency table

|  | Early respondents | Late respondents |
| --- | --- | --- |
| The students always or almost always choose the topics themselves | 64 | 45 |
| The students sometimes choose their topics, and sometimes I give them the topic that they will be working on | 215 | 83 |
| I always or almost always give them the topic that they will be working on | 209 | 95 |
| Other (please specify) | 10 | 2 |

Fisher’s Exact Test

P=0.11
