# Supplementary material for: China’s science, technology, engineering, and mathematics (STEM) research environment: A snapshot
Source: PLoS One. 2018 Apr 3;13(4):e0195347. doi: 10.1371/journal.pone.0195347 (PMC5882148; doi:10.1371/journal.pone.0195347)
Supplement: S2 Appendix — (DOC) [file pone.0195347.s002.doc]

**Supporting information**

**S2 Appendix. Survey introductory letter in Mandarin Chinese and English**

**Mandarin Chinese translation**

尊敬的教授:

我们是来自美国加州大学圣塔巴巴拉分校和亚利桑那州立大学,以及英国诺丁汉大学的研究人员。我们主要研究中国,拉丁美洲及欧洲的科技和创新架构。本研究由美国国家科学基金会资助的加州大学圣塔巴巴拉分校纳米科技与社会研究中心(www.cns.ucsb.edu)主持进行。

我们现阶段的研究目标是对中国高等院校总体科研环境及文化进行网上问卷调查。我们的调查对象是在中国排名前25位的高等院校理工院系中从事教学科研工作的教授及副教授群体。我们计划将有关中国科研群体现状综合评估的研究成果发表在*科学（Science）*及*自然 （Nature）*等高影响力期刊。您的参与将为我们提供宝贵的信息来源。因此，我们将不胜感激。

我们诚邀您参与我们关于中国高等教育科研文化的网上问卷调查。完成整个问卷大约需时15-20分钟。问卷内容包括您的科研活动，资金来源，以及您对中国科研环境的看法。您可以通过点击此个人链接：[personal link here]

以下是本研究项目带头人的简介：

理查德·阿贝尔鲍姆（Richard Appelbaum）：阿贝尔鲍姆博士是加州大学圣塔巴巴拉分校全球与国际问题研究系及社会学系研究教授和麦克阿瑟基金会（MacArthur Foundation）主席，美国国家科学基金会资助的该校纳米科技与社会研究中心主持人之一。同时，他曾经担任该校社会学系系主任及全球与国际问题研究系代理主任，他还是全球与国际问题研究系创办人之一。他拥有哥伦比亚大学学士学位，普林斯顿大学伍德罗·威尔逊公共和国际事务学院公共行政硕士学位，以及芝加哥大学博士学位。阿贝尔鲍姆教授已经发表了大量关于新兴科技（尤其是在中国的）发展，商业全球化，劳工群体生态，社会理论，城市社会学，以及公共政策的研究论著。

丹尼斯•西蒙（Denis Simon）：西蒙博士是亚利桑那州立大学校长中国问题高级顾问，也是该校政治学与全球研究系的杰出教授（Foundation Professor）。西蒙教授是世界公认的中国科技创新状况研究权威。他曾作为中国科技发展顾问为联合国，世界银行，经合组织，前美国国会科技评估办公室，美国国家科学院以及美国国家科学基金会服务。他还作为美方专家组成员参与了中国国家科技部和美国白宫科技政策办公室之间进行的“中美创新对话”。同时，他还担任了*科学*（*Science*）杂志的评审编辑。2006年，西蒙教授荣获由中国温家宝总理颁发的中国国家友谊奖并被授予中国大连荣誉市民称号。西蒙教授拥有纽约州立大学新帕尔茨分校学士学位及加州大学伯克利分校硕士及博士学位。

曹聪（Cong Cao）：曹博士是研究中国科技创新状况的著名学者。目前，他在英国诺丁汉大学当代中国研究学院任副教授。曹教授的研究兴趣包括中国科技政策制订过程中政府，科学家，企业，媒体及公众的作用和地位；科技人才，高等教育，及中国学生人才外流状况；中国与其他国家在科技体制方面的异同；以及中国作为科技超级大国的崛起。曹博士拥有哥伦比亚大学博士学位。他撰写了关于中国科学院院士的《中国科技精英》（*China's Scientific Elite* ）(2004)一书。 而另一部和丹尼斯•西蒙教授合作的著作《中国的新兴技术优势：评估高端人才的作用》（*China's Emerging Technological Edge: Assessing the Role of High-End Talent* ）（2009)则是近四十年来首部关于中国科技领域人力资源的数量，质量，及有效利用等关键性问题的研究论著.

我们真诚地希望您能够参与我们的研究。如有任何疑问，请联系阿贝尔鲍姆博士 [email address]。谢谢！

此致,

Professor Richard P. Appelbaum

MacArthur Chair in Sociology and

Global & International Studies

Co-PI, Center for Nanotechnology and Society

University of California at Santa Barbara

Santa Barbara, CA 93106-7065

Professor Denis F. Simon

Senior Advisor for China and Global Affairs to the President, Arizona State University

Foundation Professor, Political Science and Global Studies

Arizona State University

Tempe, AZ 85281

Professor Cong Cao

Associate Professor and Reader at the School of Contemporary Chinese Studies

University of Nottingham

University Park

Nottingham, UK NG7 2RD

**English translation**

Dear Professor,

We are researchers from the University of California at Santa Barbara, Arizona State University, and University of Nottingham studying science, technology, and innovation frameworks across China, Latin America, and Europe. We are currently conducting an online survey to gain a comprehensive understanding of the research environment and culture at Chinese institutions of higher education. We are contacting all Science, Technology, Engineering, and Mathematics (STEM) professors and associate professors from the top 25 ranked institutions in China to participate in our study. Your responses will remain confidential, and will not be associated in any way with your identity. We plan to publish the findings from this study in a high-impact journal such as Science or Nature to provide a comprehensive assessment of the research community in China. Your participation in this study would be greatly appreciated and provide invaluable information to our research.

We kindly ask for your participation in our online survey regarding the research culture at Chinese institutions of higher education. The survey will last approximately 15-20 minutes and includes questions about your research, funding, and your perceptions of the research environment in China. You can access your personal link to the survey here: [personal link here]

Please find a brief description of our research leadership below:

**Richard Appelbaum**, Ph.D., is MacArthur Chair and Research Professor in Global and International Studies and Sociology at the University of California at Santa Barbara, where he is co-PI at the NSF-funded Center for Nanotechnology in Society.  He has previously served as chair of the Sociology Department, and was founder and Acting Director of the UCSB Global & International Studies Program. He received his B.A. from Columbia University, M.P.A. from Princeton University's Woodrow Wilson School of Public and International Affairs, and Ph.D. from the University of Chicago. Professor Appelbaum has published extensively in the areas of emerging technologies, particularly in China; the globalization of business; the sociology of work and labor; and social theory, urban sociology, and public policy.

**Denis Simon**, Ph.D., is Senior Advisor for China and Global Affairs to the President of Arizona State University, where he is Foundation Professor in the Departments of Political Science and Global Studies. Professor Simon is recognized as one of the world’s leading experts on science, technology, and innovation in China. He has served as an adviser to the United Nations, World Bank, OECD, the former Congressional Office of Technology Assessment, the National Academy of Sciences, and the National Science Foundation regarding S&T development in the People’s Republic of China. Currently, he serves as a member of the American Experts Group as part of the U.S.-China Innovation Dialogues under the White House Office of Science and Technology Policy and China’s Ministry of Science and Technology. He is also serving on the board of reviewing editors for Science. In 2006, Dr. Simon received the China National Friendship Award from Premier Wen Jiabao and was also made an honorary citizen of the city of Dalian. Professor Simon received his B.A. from SUNY New Paltz, M.A. and Ph.D. from the University of California at Berkeley.

**Cong Cao**, Ph.D., is a leading scholar in the studies of China’s science, technology, and innovation. He is currently an Associate Professor and Reader at the School of Contemporary Chinese Studies at the University of Nottingham. Professor Cao research interests include the role of government, scientists, corporations, media and the public in the formation of science and technology policy in China; technical talent, higher education and the ‘brain drain’ of Chinese students; comparison of innovation systems in China with other countries, and China’s rise as a scientific superpower. Dr. Cao received his Ph.D. from Columbia University. He is the author of China's Scientific Elite (2004), a study of those Chinese scientists holding the elite membership in the Chinese Academy of Sciences, and China's Emerging Technological Edge: Assessing the Role of High-End Talent (with Denis Simon, 2009), the first in forty years to address these critical issues surrounding the quality, quantity, and effective utilization of China's human resources in science and technology.

We hope that you will participate in our study. Should you have any questions about this research project, please contact Dr. Appelbaum at [email address].

Sincerely,

Professor Richard P. Appelbaum

MacArthur Chair in Sociology and

Global & International Studies

Co-PI, Center for Nanotechnology and Society

University of California at Santa Barbara

Santa Barbara, CA 93106-7065

Professor Denis F. Simon

Senior Advisor for China and Global Affairs to the President, Arizona State University

Foundation Professor, Political Science and Global Studies

Arizona State University

Tempe, AZ 85281

Professor Cong Cao

Associate Professor and Reader at the School of Contemporary Chinese Studies

University of Nottingham

University Park

Nottingham, UK NG7 2RD
